# Marine Bromotyrosine Derivatives in Spotlight: Bringing Discoveries and Biological Significance

**DOI:** 10.3390/md22030132

**Published:** 2024-03-14

**Authors:** Paula Ferreira Montenegro, Giang Nam Pham, Fatouma Mohamed Abdoul-Latif, Elisabeth Taffin-de-Givenchy, Mohamed Mehiri

**Affiliations:** 1 Marine Natural Products Team, Institut de Chimie de Nice, Université Côte d’Azur, CNRS UMR 7272, 06108 Nice, France; paula.ferreira-montenegro@univ-cotedazur.fr (P.F.M.); giang-nam.pham@univ-cotedazur.fr (G.N.P.); elisabeth.taffin-de-givenchy@univ-cotedazur.fr (E.T.-d.-G.); 2 Medicinal Research Institute, Center for Studies and Research of Djibouti, IRM-CERD, Route de l’Aéroport, Haramous, Djibouti City P.O. Box 486, Djibouti; fatouma_abdoulatif@yahoo.fr

**Keywords:** marine sponges, Verongida, bromotyrosine derivatives, anticancer activities, antimicrobial activities

## Abstract

The Verongida order comprises several sponge families, such as Aplysinellidae, Aplysinidae, Ianthellidae, and Pseudoceratinidae, reported for producing bromotyrosine-derived compounds. First identified in 1913, bromotyrosine derivatives have since captivated interest notably for their antitumor and antimicrobial properties. To date, over 360 bromotyrosine derivatives have been reported. Our review focuses specifically on bromotyrosine derivatives newly reported from 2004 to 2023, by summarizing current knowledge about their chemical diversity and their biological activities.

## 1. Introduction

Marine sponges that fall under the Verongida order are categorized into four distinct families: Aplysinellidae, Aplysinidae, Ianthellidae, and Pseudoceratinidae. Among these families, Pseudoceratinidae has garnered considerable attention in the field of chemical research due to the revelation of a shared characteristic among sponges in this order—the production of bromotyrosine derivatives. Bromotyrosine is a chemical compound derived from the amino acid tyrosine, which has undergone bromination, resulting in the incorporation of one or more bromine atoms into its structure. To date, over 360 metabolites that belong to the bromotyrosine family have been identified. These metabolites are primarily found in sponges belonging to the Verongida order. Interestingly, bromotyrosines have also been detected in sponges from other taxonomic orders including Agelasida, Suberitida, Poecilosclerida, Dictyoceratida, Tetractinellida, Axinellida, and Tethyida [1]. Additionally, certain ascidians and molluscs have been identified as producers of bromotyrosines, indicating their widespread presence among marine organisms [2].

These bromotyrosines were initially documented in 1913 and were acknowledged for their noteworthy biological properties, which include antitumor, antifouling, and biocidal effects [3]. They can be classified into six main categories: simple bromotyrosines, spirocyclohexadienylisoxazolines, spirooxepinisoxazolines, oximes, bastadins, hemibastadins, and other derivatives such as cyclodepsipeptides, for instance [4]. Excluding the compounds that are classified as simple bromotyrosines, consisting of only one bromotyrosine unit, the metabolites found in the remaining categories are composed of one to four bromotyrosine units. Interestingly, there is a lower prevalence of metabolites containing four bromotyrosine units compared to those with one or two units.

**Figure 1:**
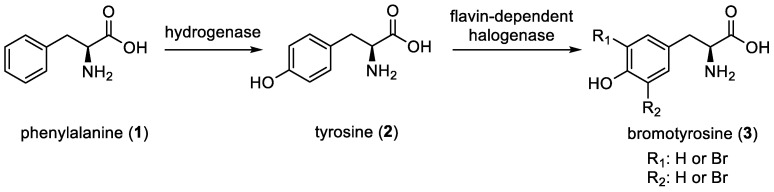
Biosynthesis of bromotyrosines by J. R. Carney and K. L. Rinehart [5].

Understanding the biosynthesis of bromotyrosines is crucial for harnessing their therapeutic potential effectively. Despite starting with basic building blocks consisting only of brominated phenylalanine (**1**) and tyrosine (**2**) units, a wide variety of derivatives are produced. They differ by the type of connections, the nature of the side chains, and the substitution patterns of the aromatic rings. In 1995, J. R. Carney and K. L. Rinehart investigated the biosynthesis of bromotyrosine derivatives by the marine sponge *Aplysina fistularis* using ^14^C-labeled amino acids such as ^14^C-*L*-Tyrosine, ^14^C-*L*-3-bromotyrosine, and ^14^C-*L*-3,5-dibromotyrosine. This study revealed that the bromotyrosine unit (**3**) of bromoisoxazoline alkaloids originated from phenylalanine, which was then converted to tyrosine (Figure 1) [5].

All the bromotyrosine derivatives isolated before 2004 were thoroughly reviewed in 2005 by J. Peng et al. [4]. Moreover, bromotyrosines have been extensively researched, with a total of 24 reviews published covering various aspects related to bromotyrosines, including their biological effects [6,7,8,9,10,11,12,13,14,15], their roles in biological processes [16], and synthetic methodologies [17]. Our review comprehensively covers newly identified bromotyrosines from 2004 to 2023, examining both their structural features and related activities. Additionally, we explore the geographical distribution of these bromotyrosines across various sponges studied, along with their associated activities.

## 2. Bromotyrosine Chemodiversity

**Figure 2:**
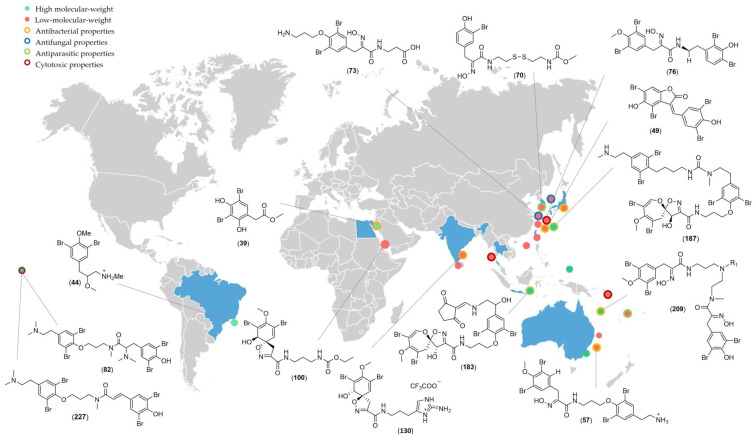
Geographical distribution of marine sponges producing bioactive bromotyrosines from 2004 to 2023.

The following part of this review is divided into two distinct sections: metabolites with a low molecular weight and those with a high molecular weight. Psammaplin A (**196**), notably its molecular weight (MW 662 Da), was used as a reference to split the bromotyrosine derivatives into the two sections mentioned above. This approach does not aim for absolute precision but rather to establish a practical distinction, which is especially important considering the diverse array of metabolites produced by marine species. Indeed, low-molecular-weight metabolites are more common compared to high-molecular-weight compounds. From 2004 to 2023, marine species reported to produce bromotyrosine derivatives were sampled mainly along the coasts of Australia [18,19,20,21,22,23,24,25,26,27], Japan [28,29,30,31,32], and India [19]. A few of them were collected along the coasts of Brazil [33] and the French Polynesian [34,35] coasts. Sponges collected along the Japanese coasts yielded mostly low-molecular-weight metabolites which exhibited a spectrum of bioactivities including antibacterial, antifungal, cytotoxic, and antiparasitic properties. Inversely, a higher prevalence of high-molecular-weight bromotyrosine derivatives, with antibacterial and antifungal properties, was observed for sponges sampled along the coasts of Australia and Indonesia. Finally, bromotyrosines reported from sponges sampled along the coasts of India predominantly had a low molecular weight and exhibited antibacterial properties (Figure 2).

### 2.1. Low-Molecular-Weight Metabolites

#### 2.1.1. Simple Bromotyrosines

**Figure 3:**
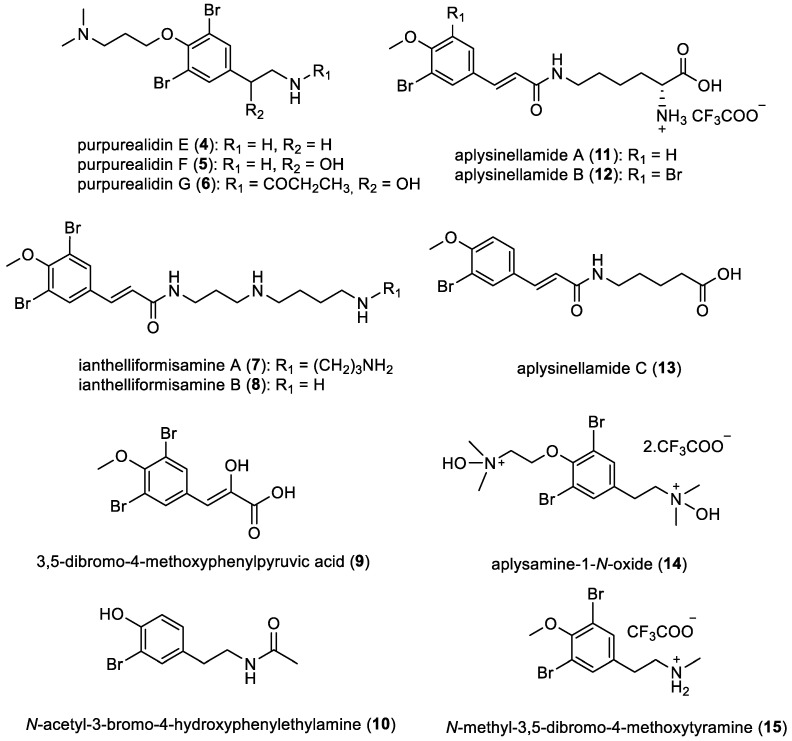
Structures of simple bromotyrosines.

Three novel simple bromotyrosines, purpurealidins E (**4**), F (**5**), and G (**6**), along with the methyl ester of *N*-((3,5-dibromo-4-(3-methylaminopropoxy)phenyl)ethyl)carbamic acid, were isolated in 2004 by S. Tilvi et al. from the sponge *Psammaplysilla purpurea* collected in Tamil Nadu, India [19]. However, the stereochemistry of purpurealidins remains undetermined. In 2012, ianthelliformisamines A (**7**) and B (**8**) were reported from the sponge *Suberea ianthelliformis* collected in North Stradbroke Island, Australia [18]. Two years later, 3,5-dibromo-4-methoxyphenylpyruvic acid (**9**) and *N*-acetyl-3-bromo-4-hydroxyphenylethylamine (**10**) were isolated from *Callyspongia* sp. (Haplosclerida order), collected in Manta Ray Bommie, North Stradbroke Island, Australia [20]. Moreover, a series of derivatives, aplysinellamines A–C (**11**–**13**) and aplysamine-1-*N*-oxide B (**14**), were isolated from the sponge *Aplysina* sp. collected in the Great Barrier Reef, Australia. These compounds were obtained as trifluoroacetic acid (TFA) salts through the purification process [21]. In addition, *N*-methyl-3,5-dibromo-4-methoxytyramine (**15**) was isolated from the sponge *Narrabeena nigra* collected from North-west ledge, Heron I, Capricorn–Bunker Group, Great Barrier Reef, Queensland, Australia (Figure 3) [22].

- Subereamides, tyrokeradines, and derivatives

**Figure 4:**
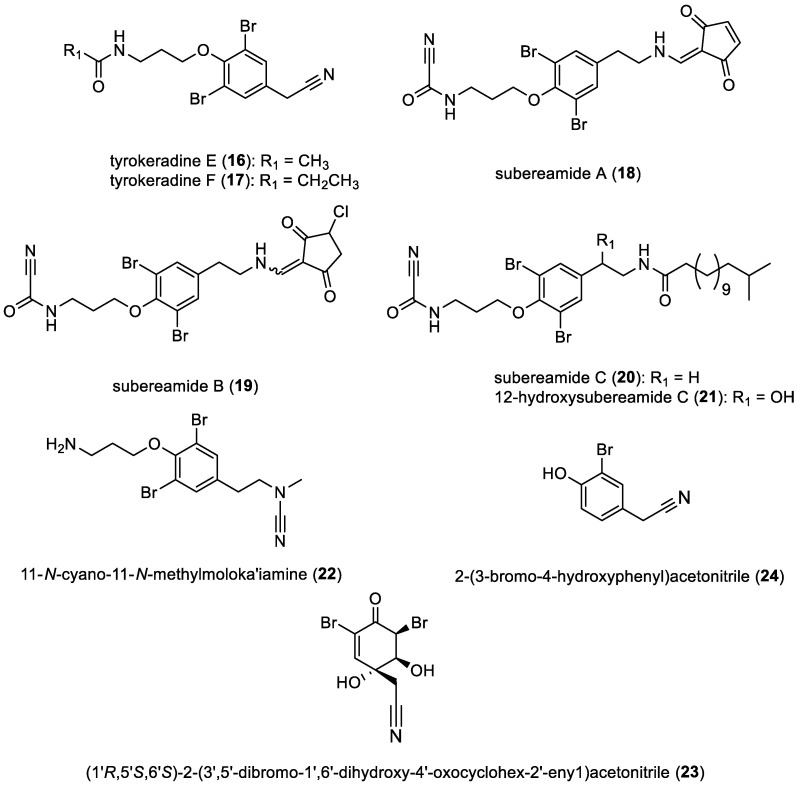
Structures of simple bromotyrosines with a cyano group.

Several single bromotyrosine derivatives featuring a cyano group have been isolated recently. These include tyrokeradines E (**16**) and F (**17**) extracted from an unidentified sponge belonging to the Verongida order [36]. Y. J. Lee et al. isolated subereamides A–C (**18**–**20**) and 12-hydroxysubereamide C (**21**) from *Suberea* sp. collected off the coast of Chuuk in the Federated States of Micronesia [37]. Subereamide B (**19**) was separated as a combination of *E*/*Z* isomers in a 1:1 ratio. Additionally, 11-*N*-cyano-11-*N*-methylmoloka′iamine (**22**) was isolated from *Hexadella* sp. [38]. Furthermore, (1’*R*,5’*S*,6’*S*)-2-(3’,5’-dibromo-1’,6’-dihydroxy-4’-oxocyclohex-2’-enyl)acetonitrile (**23**) was isolated from the sponge *Pseudoceratina* sp. [39]. In 1987, 2-(3-bromo-4-hydroxyphenyl)acetonitrile (**24**) was isolated from an unidentified sponge [40]. More recently, in 2020, (**24**) was re-isolated from the Fijian marine sponge *Aplysinella rhax* by E. T. Oluwabusola et al. [41]. All compounds are represented in Figure 4.

- Ma’edamines C–F

**Figure 5:**
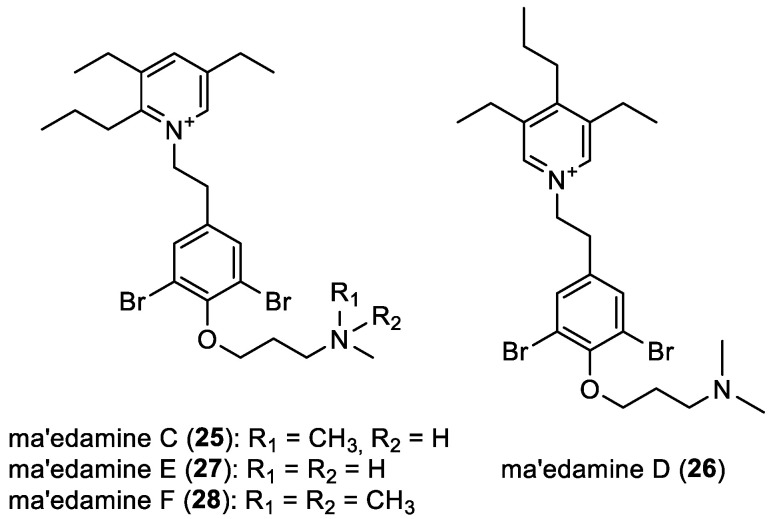
Structures of ma’edamines C–F (**25**–**28**).

In 2019, S. Kurimoto et al. isolated ma’edamines C (**25**) and D (**26**) from the marine sponge *Suberea* sp. collected in Maeda Cape, Okinawa, Japan, marking the first identification of natural compounds featuring a tetrasubstituted pyridinium moiety [28]. In 2022, ma’edamines E (**27**) and F (**28**), unusual natural products featuring a 1,2,3,5-tetrasubstituted pyridinium moiety, were obtained from the same source (Figure 5) [42].

- Other simple bromotyrosines

**Figure 6:**
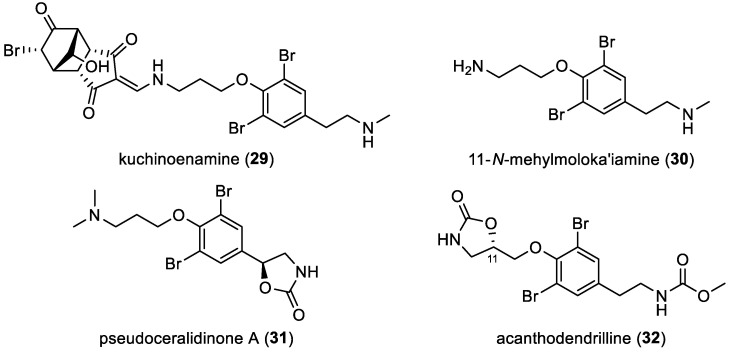
Structures of other simple bromotyrosines.

A chemical investigation of the marine sponge *Hexadella* sp. by S. Matsunaga et al. led to the isolation of a new metabolite, kuchinoenamine (**29**), featuring a tricyclo [5.2.1.0]-decane subunit linked to 11-*N*-methylmoloka’iamine (**30**) through an enamine bond [38]. Kuchinoenamine (**29**) is biogenetically related to psammaplysin E (**174**) [43]. In fact, the tricyclo [5.2.1.0^2,6^]-decane moiety may be formed by a [4 + 2] cycloaddition of a cyclopenta-2,4-dienol and an *N*-substituted 2-aminomethylenecyclopent-4-ene-1,3-dione followed by bromohydrin formation and oxidation. Pseudoceralidinone A (**31**), a bromotyrosine-derived compound featuring an oxazolidone motif, was successfully isolated from the sponge *Pseudoceratina verrucose* [44]. Subsequently, another derivative, reported as acanthodendrilline (**32**), was also identified from the sponge *Acanthodendrilla* sp. The absolute configuration of C-11 within (**32**) was determined to be *S* through the total synthesis of both enantiomers and a comparison of the optical properties of the synthetic and natural compounds (Figure 6) [45].

Synoxazolidinones A (**33**) and C (**34**), along with pulmonarins A (**35**) and B (**36**), were successfully isolated by R. Trepos et al. from the ascidian species *Synoicum pulmonaria* [46].

In 2009, the isolation of aphrocallistin (**37**) was reported, originating from the sponge *Aphrocallistes Beatrix* [47]. Indeed, (**37**) is an unusual bromotyrosine compound as it features an adenine subunit. Furthermore, in 2015, aplysinin B (**38**), featuring a distinctive 2-aminoimidazole motif, was extracted from the sponge *Aplysina lacunose* [48]. Additional derivatives falling into the dibromotyrosine category include subereaphenols B (**39**) and C (**40**), which were sourced from the sponge *Suberea mollis* collected in the Red Sea by M. I. Abou-Shoer et al. [49].

In 2010, agelanesins A (**41**) and C (**42**) were isolated from two Indonesian *Agelas* sponges. They feature an unprecedented combination of a brominated tyramine unit and a pyrrole unit [50]. Additionally, 2-(3-amino-2,4-dibromo-6-hydroxyphenyl)acetic acid (**43**) was isolated from the sponge *Aplysina cauliformis*, while 3-(3,5-dibromo-4-methoxyphenyl)-2-methoxy-*N*-methylpropan-1-ammonium (**44**) was recovered from the sponge *Pachychalina* sp., collected in Ilha do Pai, Niteroi, Rio de Janeiro state, Brazil [33]. Lastly, 2-(3,5-dibromo-4-hydroxyphenyl)-*N*,*N*,*N*-trimethylethan-1-aminium (**45**) and 2-(3,5-dibromo-4-methoxyphenyl)-*N*,*N*,*N*-trimethylethan-1-aminium (**46**) were isolated from the sponge *Verongula rigida* [51].

**Figure 7:**
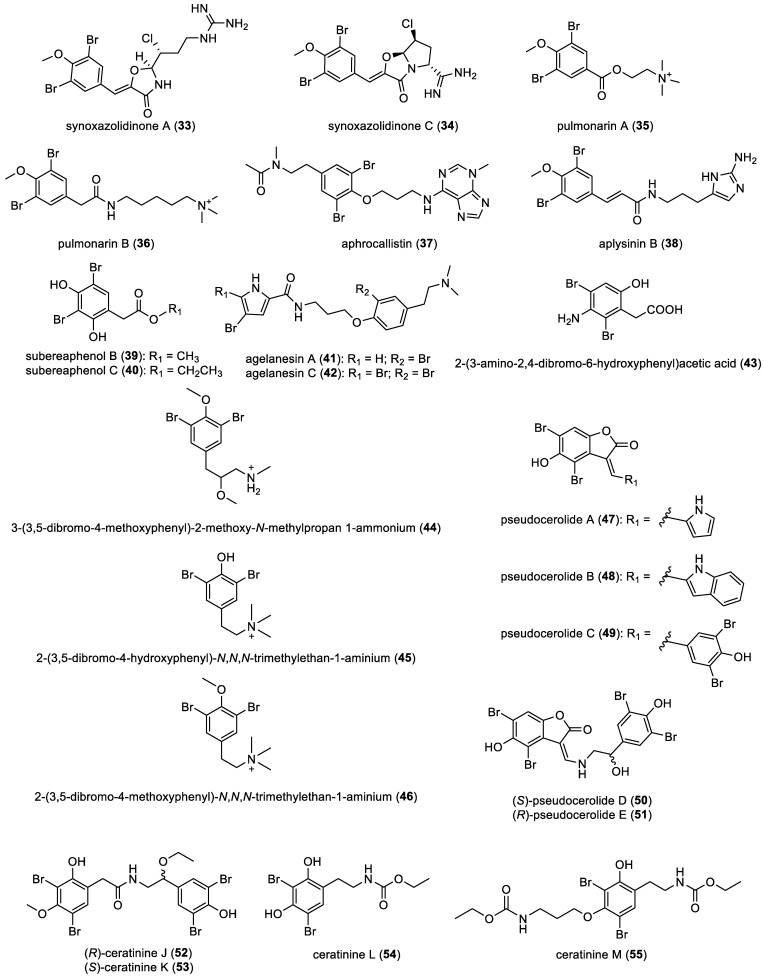
Structure of simple bromotyrosines.

Pseudocerolides A–E (**47**–**51**) were isolated from the marine sponge *Pseudoceratina* sp. collected in the South China Sea and represent a distinctive group of bromotyrosine derivatives that share a common structural core featuring a dibrominated benzofuranone unit. Each compound within this group possesses distinct side chains, differentiating them from each other [41,42,43]. Several other derivatives, including ceratinines J–M (**52**–**55**), were also extracted from the same sponge [52]. All compounds are represented in Figure 7.

#### 2.1.2. Oximes

This class comprises oximes with a histamine unit, a bromotyramine unit, a disulfide bond, or other, rarer unit(s).

- Aplysamines, aplyzanzines, and purpuramines

**Figure 8:**
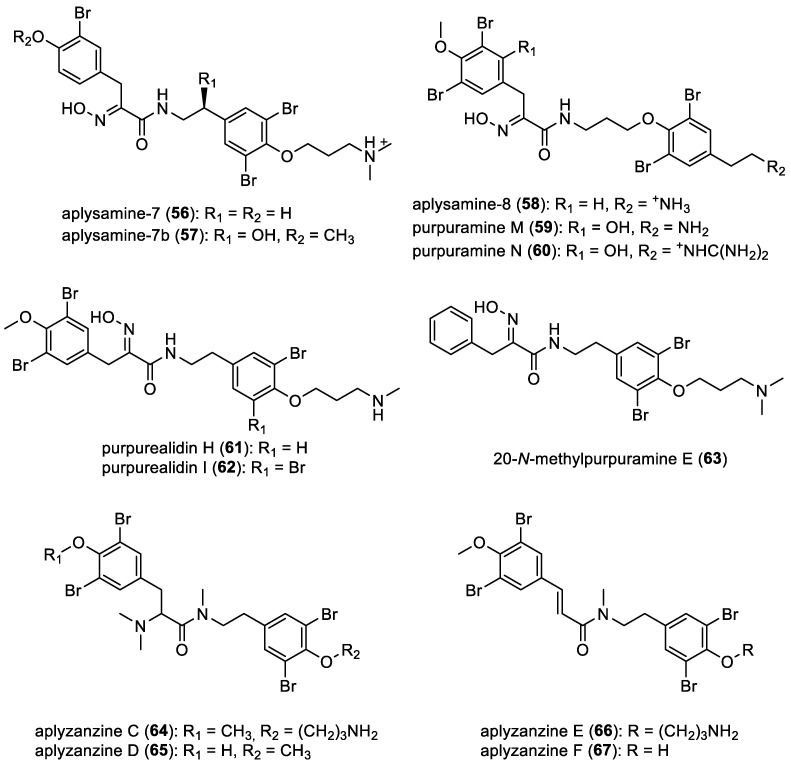
Structures of oximes with a bromotyrosine unit.

Aplysamine-7 (**56**), originally isolated from *Suberea ianthelliformis* collected in the Solomon Islands, was also isolated from *Pseudoceratina* spp. [23]. Another derivative, reported as “aplysamine-7b” (**57**), and aplysamine-8 (**58**) were identified in *Pseudoceratina purpurea* and its predator, *Tylodina corticalis* [44,53]. Furthermore, in 2016, purpuramines M (**59**) and N (**60**) were isolated from a sponge belonging to the family Aplysinellidae from Manta Point, Sangalaki, Indonesia [54]. From the sponge *Psammaplysilla purpurea*, purpurealidin H (**61**) was isolated and characterized. On the other hand, purpurealidin I (**62**), was identified only by tandem mass spectrometry [18,55]. Furthermore, 20-*N*-methylpurpuramine E (**63**) was isolated from *Pseudoceratina purpurea* collected off Bise, Okinawa, Japan. This bromotyrosine derivative is particular due to the absence of both alcohol and bromine on the aromatic ring adjacent to the oxime moiety [29]. In 2020, four novel brominated tyrosine metabolites, designated as aplyzanzines C–F (**64**–**67**), were isolated from the sponge species *Pseudoceratina* sp. originating from French Polynesia [34]. All compounds are represented in Figure 8.

- Psammaplins C–D and O–P

**Figure 9:**
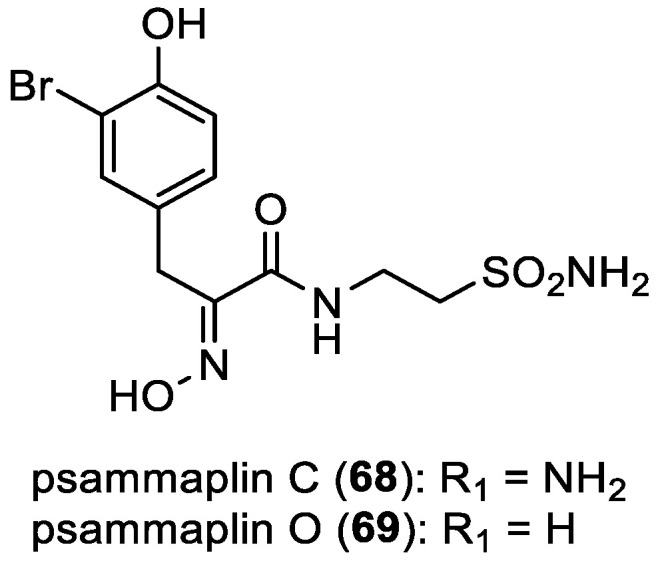
Structures of psammaplins C (**68**) and O (**69**).

Psammaplin C (**68**) was originally isolated from the marine sponge *Psammaplysilla purpurea* by C. Jimenez and P. Crews in 1990 [56]. Subsequently, in 2020, E. T. Oluwabusola et al. isolated psammaplin O (**69**), along with psammaplin C (**68**), from the Fijian marine sponge *Aplysinella rhax* (Figure 9) [52].

**Figure 10:**
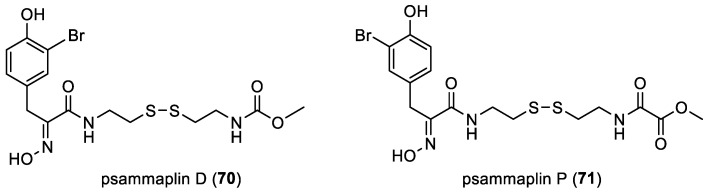
Structures of psammaplins D (**70**) and P (**71**).

In 2003, psammaplin D (**70**) was isolated by Y. Park et al. from the association of two sponges, *Jaspis wondoensis* and *Poecillastra wondoensis*, collected off the coast of Gomun Island, Korea [57]. In 2020, E. T. Oluwabusola et al. isolated psammaplin P (**71**) along with the already reported psammaplin D (**70**) from the marine sponge *Aplysinella rhax* collected from the Fiji islands (Figure 10) [41].

- Psammaplins and tyrokeradines

**Figure 11:**
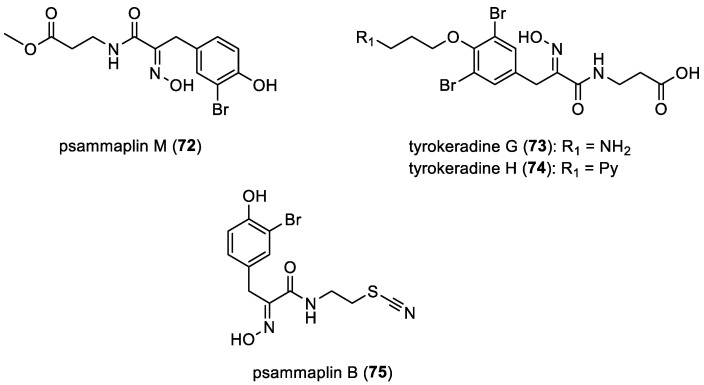
Structures of other oxime bromotyrosines.

Psammaplin M (**72**) was isolated in 2008 from the symbiotic association of the sponges *Jaspis* sp. and *Poecillastra* sp. [58]. In 2015, psammaplin M (**72**) and tyrokeradines G (**73**) and H (**74**) were isolated from a sponge of the order Verongida [36]. These oximes are composed of a *β*-alanine moiety and a tyrokeradine unit, wherein tyrokeradine H (**74**) features an extra pyridinium unit. Psammaplin B (**75**) was isolated for the first time in 1991 by C. Jimenez and P. Crews from the marine sponge *Psammaplysilla purpurea* [56]. Recently, in 2020, psammaplin B (**75**) was also isolated by E. T. Oluwabusola et al. from the Fijian marine sponge *Aplysinella rhax* [41]. The SCN functional group is distinctive and does not seem to have analogous counterparts among any known amino acid derivatives found in marine sponges (Figure 11) [56].

#### 2.1.3. Hemibastadin Derivatives

**Figure 12:**
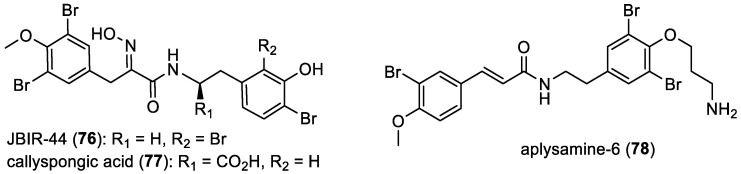
Structures of JBIR-44 (**76**), callyspongic acid (**77**), and aplysamine-6 (**78**).

Since 2004, several bromotyrosine derivatives closely related to hemibastadins have been isolated. JBIR-44 (**76**) represents the initial example of such compounds [59]. Subsequently, another derivative, callyspongic acid (**77**), was re-isolated from a species of *Callyspongia*, collected off the coast of Australia [20]. Finally, aplysamine-6 (**78**), which exhibits an additional double bond compared to hemibastadins, was also isolated from the sponge *Pseudoceratina* sp. sampled in North Halls off Sunshine Coast, Queensland, Australia (Figure 12) [24].

### 2.2. High-Molecular-Weight Metabolites

#### 2.2.1. Anomoians A–F and Ianthelliformisamine C

**Figure 13:**
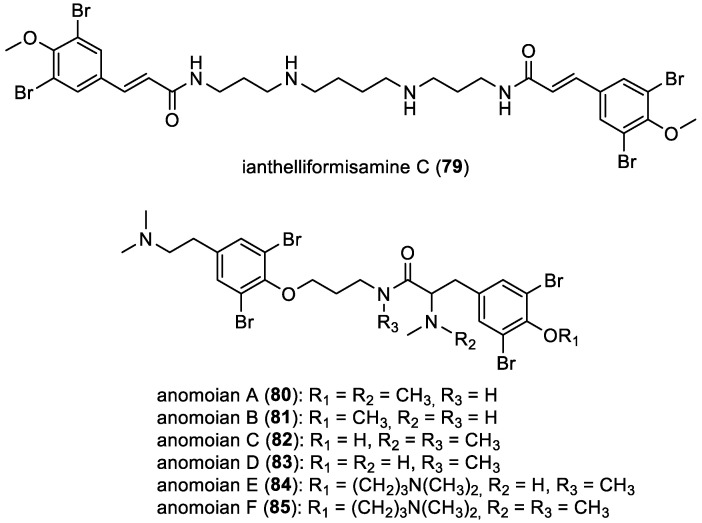
Structures of ianthelliformisamine C (**79**) and anomoians A–F (**80**–**85**).

In 2012, ianthelliformisamine C (**79**) was reported from the sponge *Suberea ianthelliformis* collected in Manta Ray Bommie, North Stradbroke Island, Australia [18]. Ianthelliformisamines A (**7**) and B (**8**) (Figure 3) are composed of a single bromotyrosine unit while ianthelliformisamine C (**79**) possesses two of these units. A similar scaffold is observed in anomoians A–F (**80**–**85**), in which two bromotyrosine units are present. Anomoian A (**80**) was initially isolated in 1990 from the marine sponge *Anomoianthella popeae* [60]. More recently, in 2017, anomoian B (**81**) was isolated from the organic extracts of a Verongida sponge belonging to the *Hexadella* genus, as well as from a two-sponge association involving *Jaspis* sp. and *Bubaris* sp. [61]. Additionally, anomoians C–F (**82**–**85**) were also isolated in 2018 from the French Polynesian sponge *Suberea ianthelliformis* (Figure 13) [35].

#### 2.2.2. Spirocyclohexadienylisoxazolines

**Figure 14:**
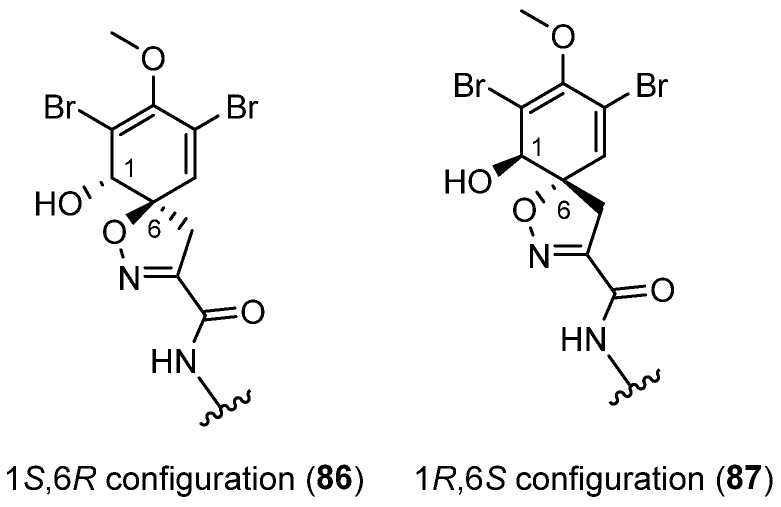
Absolute configurations of spirocyclohexadienylisoxazolines moieties.

This category comprises two main subclasses: *mono*-spirocyclohexadienylisoxazolines and *bis*-spirocyclohexadienylisoxazolines. The latter subclass accommodates various structural features, including the presence of a bromotyrosine unit, a histamine unit, a linear side chain, or a combination of these constituent units. The absolute configuration of the stereogenic centers of the spiroisoxazoline moiety within spirocyclohexadienylisoxazolines has been determined to be 1*S*,6*R* (**86**) or 1*R*,6*S* (**87**). Most of these alkaloid derivatives exhibit a 1*R*,6*S* configuration (**87**) (Figure 14).

**Figure 15:**
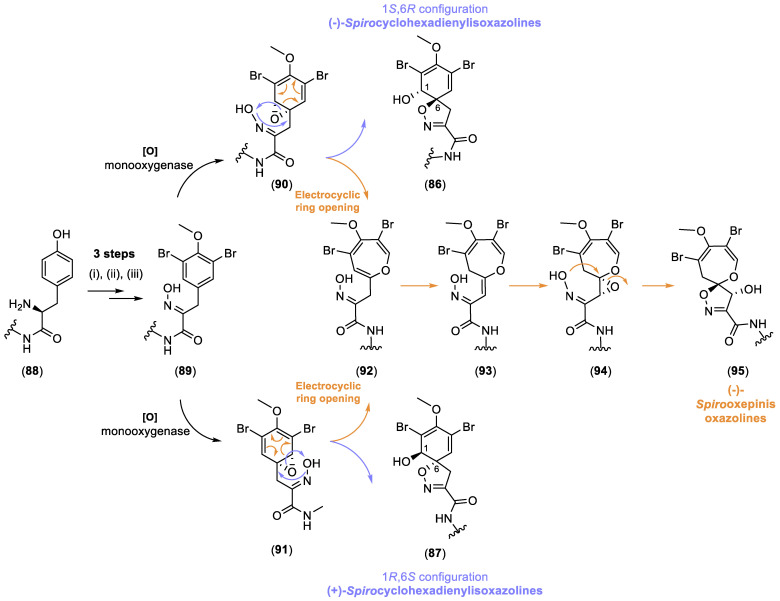
Proposed biogenesis of spirocyclohexadienylisoxazoline bromotyrosine alkaloids by K. Ragini et al. [25].

The biosynthesis of this class of alkaloids, as proposed by Ragini et al., involves an enantiodivergent step, resulting in structural diversity [25]. The dearomatization step, depicted in Figure 15, is likely the key enantiodivergent step where this transformation occurs, facilitated by a monooxygenase, possibly cytochrome P45030.

Tyrosine (**88**) undergoes a series of enzymatic reactions: (i) a deamination catalyzed by an aminotransferase, (ii) a methylation via a methylase, a bromination through a bromoperoxidase, and (iii) a conversion of the keto group facilitated by an oximinotransferase, resulting in the formation of an oxime (**89**). Subsequently, for the generation of the spiroisoxazoline moiety, it has been proposed that a monooxygenase catalyzes the epoxidation of the aromatic ring (**90**) and (**91**), which can then cyclize to yield the isooxazolines (**86**) and (**87**). This process of enantiotopic epoxidation leads to an intriguing enantiodivergent desymmetrization. The formation of oxepin from the common epoxide intermediate occurs through an enantioconvergent thermally allowed disrotatory electrocyclic ring opening, followed by a 1,3-sigmatropic hydride shift and a subsequent epoxidation, which is captured by the oxime to yield the dihydrooxepins (**95**) (Figure 15) [25].

##### *Mono*-spirocyclohexadienylisoxazolines

- (+/−)-Purealidin R, lacunosins A–B, subereamollines A–B, and purpuroceratic acids A–B

**Figure 16:**
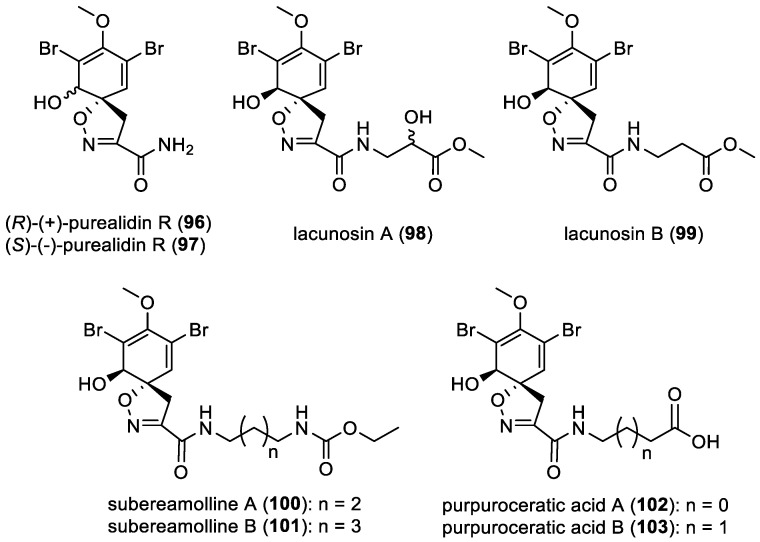
Structures of *mono*-spirocyclohexadienylisoxazolines with a side chain.

Since 2004, only a few *mono*-spirocyclohexadienylisoxazolines containing a single bromotyrosine unit were reported. In 2011, E. Galeano et al. isolated (*R*)-(+)-purealidin R (**96**) from *Psammaplysilla purpurea* (previously *Pseudoceratina purpurea*) which was re-isolated from the marine sponge *Verongula rigida* [51,62]. One year later, (*S*)-(−)-purealidin R (**97**) was isolated from *Pseudoceratina* spp. collected in Port Campbell, Victoria, Australia [23]. More recently, N. Mariam et al. isolated lacunosin A (**98**) and B (**99**) from the Caribbean marine sponge *Aplysina lacunosa*. Lacunosins A–B (**98**–**99**) are coupled to a glycine and an isoserine methyl ester, respectively [63]. Moreover, subereamollines A–B (**100**–**101**), isolated from *Suberea mollis* collected in the Red Sea, and purpuroceratic acids A–B (**102**–**103**), isolated from *Pseudoceratina purpurea*, represent the only examples of *mono*-spirocyclohexadienylisoxazolines with a side chain (Figure 16) [49,64].

- Araplysillins, ianthesin E, purealidins, purpurealidins, and pseudoceratinamides

A group of derivatives reported as purpurealidins A–D (**104**–**107**) and J (**108**) were isolated from *Psammaplysilla purpurea* [55]. While purpurealidins A–D (**104**–**107**) were successfully isolated and extensively characterized, purpurealidin J (**108**) was deducted from tandem mass spectrometry analyses. In 2008, purealidins T (**109**) and U (**110**) were also identified following their extraction from the sponge *Pseudoceratina* sp., collected from the coastline of southern Sanya, Hainan Island, China [65].

**Figure 17:**
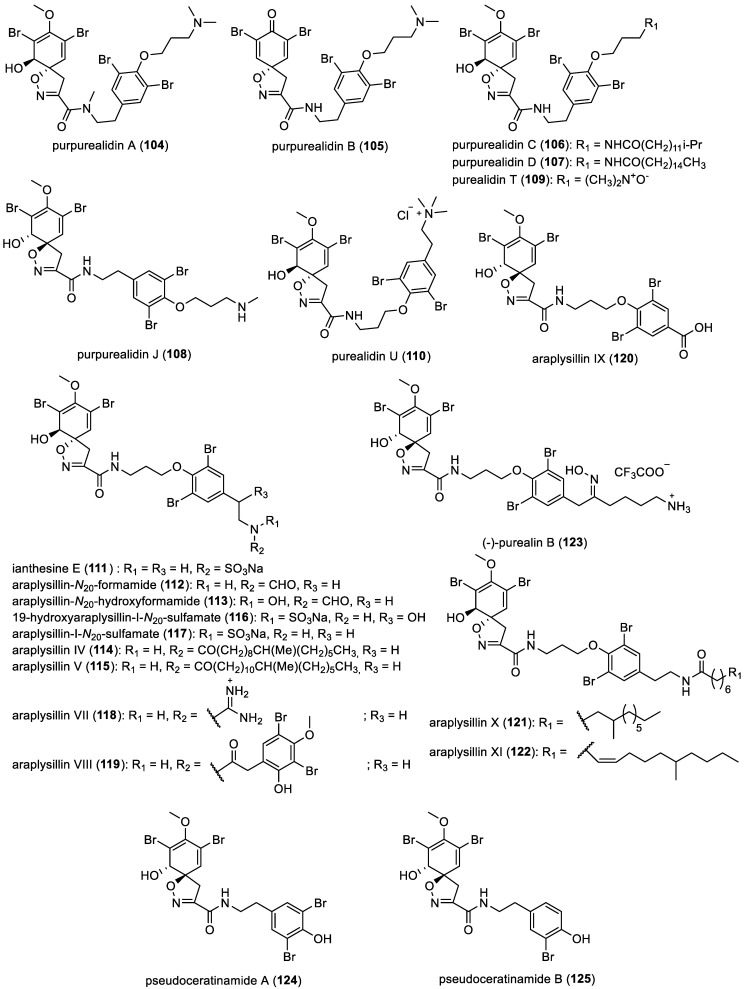
Structures of *mono*-spirocyclohexadienylisoxazolines with a bromotyrosine unit.

Ianthesin E (**111**), featuring a terminal sulfate group, was obtained from *Pseudoceratina* sp. [66]. Several araplysillin analogs, including araplysillin-*N*_20_-formamide (**112**), araplysillin-*N*_20_-hydroxyformamide (**113**), and araplysillins IV (**114**) and V (**115**), were isolated from the sponge *Suberea ianthelliformis* [67]. Moreover, in 2009, two araplysillin derivatives with a sulfamate group, 19-hydroxyaraplysillin-I-*N*_20_-sulfamate (**116**) and araplysillin-I-*N*_20_-sulfamate (**117**), were recovered from the sponge *Ianthella flabelliformis* [68]. Additionally, araplysillins VII–XI (**118**–**122**) were identified from an Indonesian sponge belonging to the family Aplysinellidae [54]. Lastly, (−)-purealin B (**123**) represents the most recent example of a bromotyrosine *mono*-spirocyclohexadienylisoxazoline, which was isolated from *Pseudoceratina* sp. in Port Campbell, Victoria, Australia. This derivative was obtained as trifluoroacetic acid (TFA) salts through the purification process [23]. In 2017, pseudoceratinamide A (**124**) and pseudoceratinamide B (**125**) were isolated from a Western Australian marine sponge, *Pseudoceratina verrucosa* (Figure 17) [25].

- Ceratinadins A–D and (*−*)-aerophobin-2

**Figure 18:**
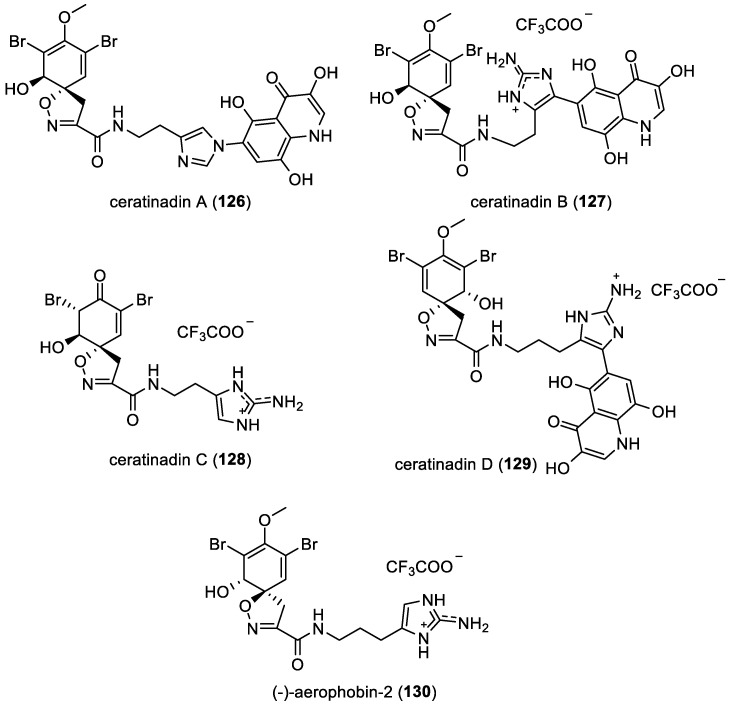
Structures of *mono*-spirocyclohexadienylisoxazolines with a histamine unit.

From 2010 to 2015, five functionalized *mono*-spirocyclohexadienylisoxazolines with histamine units, ceratinadins A–C (**126**–**128**), ceratinadin D (**129**), and (*−*)-aerophobin-2 (**130**) were reported. Ceratinadins A–C (**126**–**128**) were isolated from the sponge *Pseudoceratina* sp. [30], while ceratinadin D (**129**) was isolated from the sponge *Pseudoceratina purpurea* and its predator, *Tylodina corticalis* [42]. Lastly, (*−*)-aerophobin-2 (**130**) was isolated from specimens of *Pseudoceratina* spp. collected in Port Campbell, Victoria, Australia [23]. It is worth noting that in addition to the histamine motif, ceratinadins A (**126**), B (**127**), and D (**129**) also feature an *N*-imidazolylquinolinone motif in their molecular structures. These compounds were obtained as trifluoroacetic acid (TFA) salts through the purification process (Figure 18).

- (+/−)-Purealin and desaminopurealin

**Figure 19:**
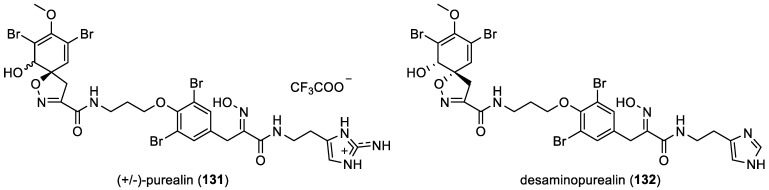
Structure of (+/−)-purealin (**131**) and desaminopurealin (**132**).

In 2012, (+/−)-purealin (**131**), a *mono*-spirocyclohexadienylisoxazoline derivative, was isolated from *Pseudoceratina* sp. as a racemic mixture (Figure 19). (+/−)-Purealin (**131**) feature a combination of a bromotyramine, an oxime, and a histamine unit in their molecular structure. The racemic mixture was obtained as a trifluoroacetic acid (TFA) salt through the purification process [23]. More recently, N. Mariam et al. isolated desaminopurealin (**132**) from the Caribbean marine sponge *Aplysina lacunosa*. Desaminopurealin (**132**) is linked, contiguously, to an *O*-1-aminopropyl 3,5-dibromotyrosyl ether and to a histamine through an amide bond [63].

##### *Bis*-spirocyclohexadienylisoxazolines

**Figure 20:**
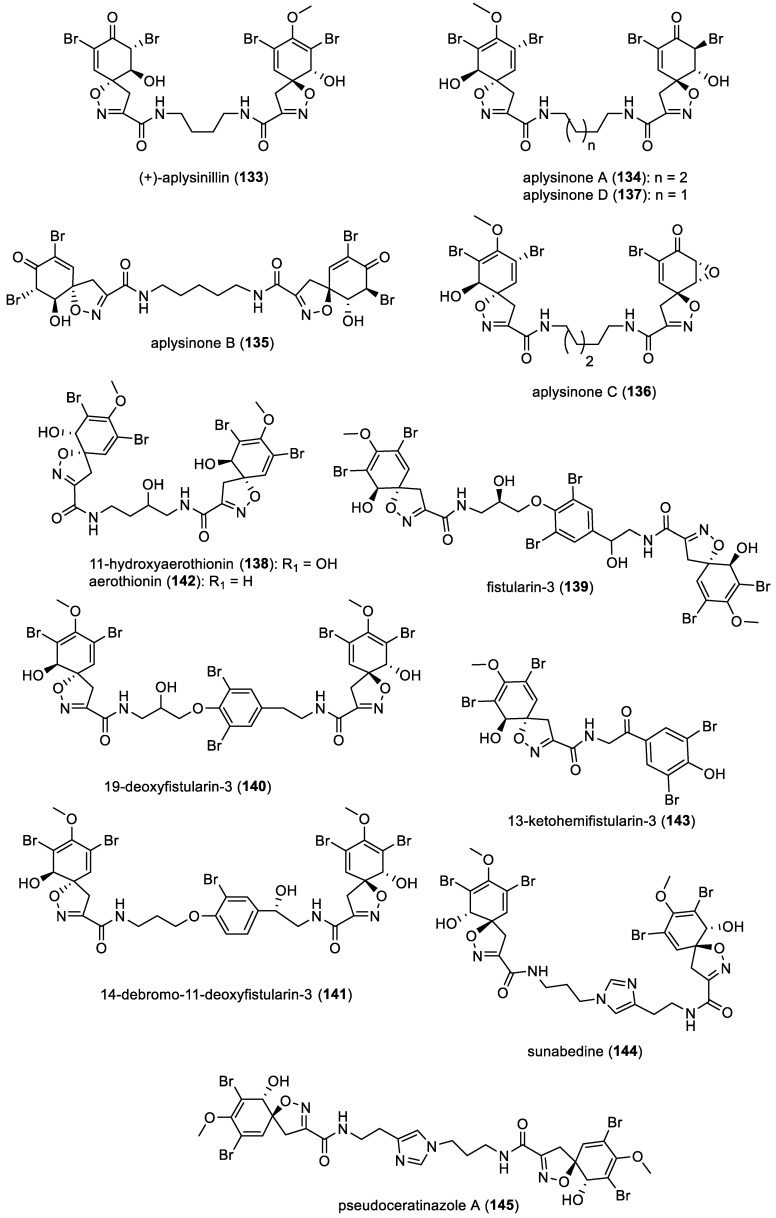
Structures of *bis*-spirocyclohexadienylisoxazolines.

Since 2004, several *bis*-spirocyclohexadienylisoxazolines were reported such as (+)-aplysinillin (**133**) from *Aplysinella* sp. [69], aplysinones A–D (**134**–**137**) from *Aplysina gerardogreeni* [70], and 11-hydroxyaerothionin (**138**) from *Verongula rigida* [51]. They are characterized by a central alkyl chain which maintains a straightforward structure. Two additional derivatives featuring a bromotyramine unit, fistularin-3 (**139**) and 19-deoxyfistularin-3 (**140**), along with the already described 14-dibromo-11-deoxyfistularin-3 (**141**), were isolated from *Aplysina lacunose* [48]. It is important to note that 11-hydroxyaerothionin (**138**) and fistularin-3 (**139**) were also isolated from cultures of the marine bacterium *Pseudovibrio denitrificans* (Ab134, isolated from the tissues of the marine sponge *Arenosclera brasiliensis*) [71]. Few studies have revealed that the chitin skeleton of sponges is tightly bound to unknown chemical structures, possibly including bromotyrosines [72]. Another study indicated the presence in skeletal fibers of several brominated derivatives such as aerothionin (**142**) as well as within spherulous cells of *Aplysina* sponges [73]. 13-ketohemifistularin-3 (**143**) was isolated from a *Pseudoceratina* sp. marine sponge collected in the South China Sea [52]. Furthermore, sunabedine (**144**), obtained from a sponge belonging to the Verongida order with an unspecified taxonomy [74], and pseudoceratinazole A (**145**), isolated from *Pseudoceratina* sp., are both *bis*-spirocyclohexadienylisoxazolines characterized by the presence of an imidazole unit in their molecular structure [75]. All compounds are represented in Figure 20.

**Figure 21:**
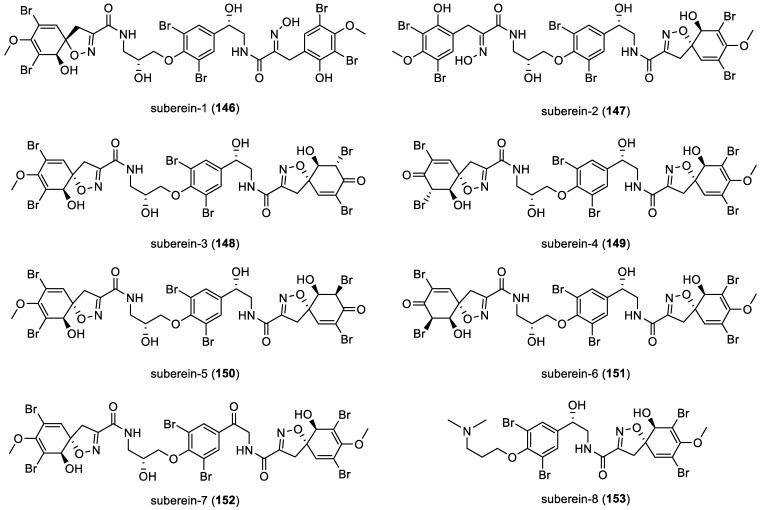
Structures of the *bis*-spirocyclohexadienylisoxazolines suberein-1 (**146**) to suberein-8 (**153**).

Moreover, in 2021, subereins 1–8 (**146**–**153**), along with twelve co-isolated compounds including fistularin (Figure 20) and subereaphenol (Figure 7) derivatives, were isolated by C. Moriou et al. from the South Pacific marine sponge *Suberea clavate* [76]. All suberein structures are represented in Figure 21.

#### 2.2.3. Spirooxepinisoxazolines

This class is constituted by spirooxepinisoxazolines containing an aminopentanedione unit and a bromotyramine unit.

- Psammaplysin derivatives

In 1982, Y. Kashman et al. isolated the first spirooxepinisoxazoline dibromotyrosine derivative, psammaplysin A (**154**), from the methanolic extract of *Psammaplysilla purpurea* collected in the southern region of the Gulf of Eilat, adjacent to the Red Sea. Psammaplysin A (**154**) was initially characterized with a proposed spiro[4.5]oxazadecane scaffold [77]. However, in 1985, D. M. Roll et al. re-isolated (**154**) from the sponge *P. purpurea* collected in Palau. Extensive NMR analyses and single-crystal X-ray diffraction studies led to a revision of the proposed structure from a spiro[4.5]oxazadecane skeleton to a spiro[4.6]dioxazundecane structure. D. M. Roll et al. highlighted the challenges in determining specific ^13^C-^13^C connections by NMR spectroscopy, particularly between C-9 and C-8 and between C-2 and C-1. However, the ketal carbon C-6 in the spiro[4.6]dioxazundecane system has a resonance by NMR spectroscopy significantly downfield compared to the spiro [5.5]dioxundecane [78]. More recently, in 2015, the definitive absolute configuration of (**154**) was determined as (6*R*,7*R*) by electronic circular dichroism (ECD), supported by time-dependent density-functional theory (TDDFT) ECD calculations, and nuclear magnetic resonance (NMR) analyses of the corresponding methoxyphenylacetic acid ester [79].

**Figure 22:**
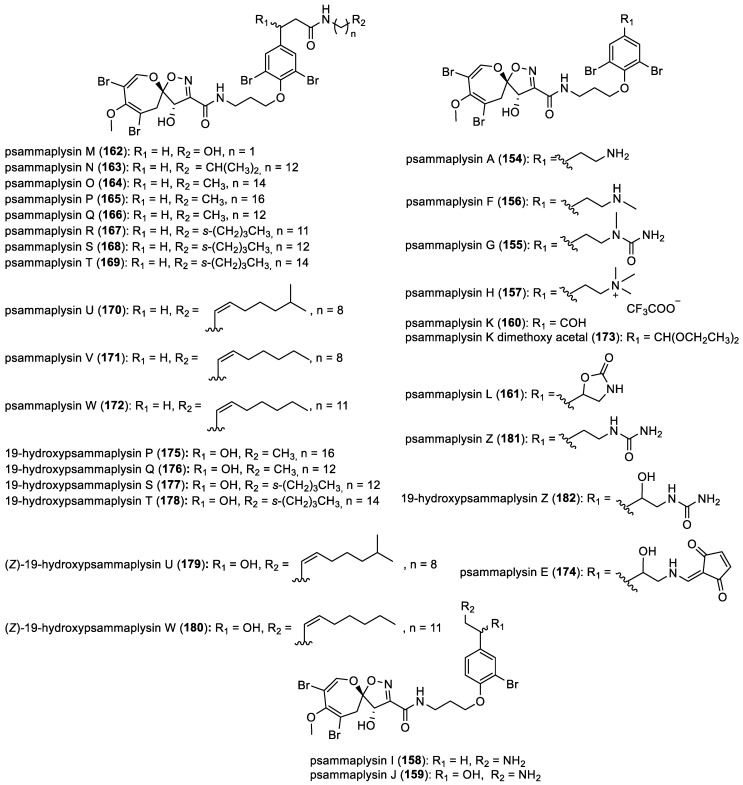
Structure of simple spirooxepinisoxazolines.

It was imperative to await the 2010s for the appearance of additional psammaplysin derivatives, characterized by a notable diversity in their chemical structures. In 2010, X. Yang et al. identified a novel bromotyrosine alkaloid, psammaplysin G (**155**), along with the previously isolated psammaplysin F (**156**), from the marine sponge *Hyatella* sp. collected from Hervey Bay, Little Woody, Sponge Garden, Queensland, Australia [26]. In fact, psammaplysin F (**156**) was isolated for the first time in 1997 by S. Liu et al. from the sponge *Aplysinella* sp. [80]. In 2011, psammaplysin H (**157**) was isolated along with the previously reported analogs, psammaplysins G (**155**) and F (**156**). These compounds were isolated from a CH_2_Cl_2_/CH_3_OH extract sourced from a marine sponge belonging to the genus *Pseudoceratina* [81]. In 2012, A. D. Wright et al. identified two new derivatives, psammaplysins I (**158**) and J (**159**), from the organic extracts of *Suberea* sp., which are examples of compounds containing a bromotyramine moiety rather than the more usual bromide analog [82]. In the same year, I. W. Mudianta isolated twenty new spirooxepinisoxazolines from a Balinese marine sponge *Aplysinella strongylata* collected in the Tulamben Bay, along with psammaplysins K–W (**160**–**172**), psammaplysin K dimethoxy acetal (**173**), and 19-hydroxypsammaplysins E (**174**), P (**175**), Q (**176**), S (**177**), T (**178**), U (**179**), and W (**180**). HPLC and Mosher ester studies confirmed that the isolated metabolites possessing a 19-OH substituent were mixtures of diastereomers [83]. In 2019, psammaplysin Z (**181**) and 19-hydroxypsammaplysin Z (**182**) were isolated with the previously reported psammaplysin A (**154**) from the methanolic extract of the Verongida Red Sea sponge *Aplysinella* sp. [84]. All compounds are represented in Figure 22.

**Figure 23:**
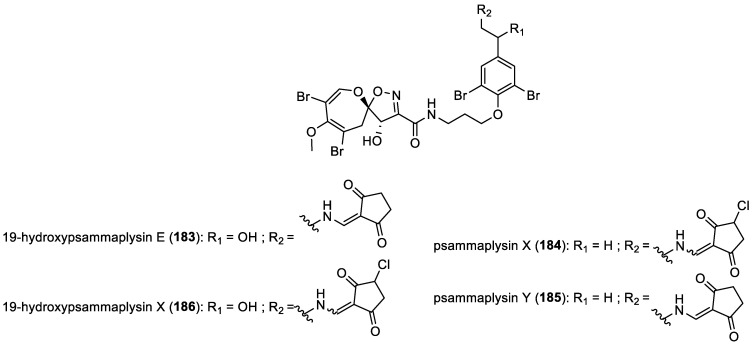
Structures of spirooxepinisoxazolines with an aminopentanedione unit.

In 2012, I. W. Mudianta et al. isolated 19-hydroxypsammaplysin E (**183**), a new bromotyrosine derivative, from the Indonesian marine sponge *Aplysinella strongylata* [83]. In 2013, Y. Lee isolated from the marine sponge *Suberea* sp. three new psammaplysin derivatives, psammaplysins X (**184**), Y (**185**), and 19-hydroxypsammaplysin X (**186**) (Figure 23), along with the already reported psammaplysin A (**154**) described just above [37].

- Ceratinadins E–F

**Figure 24:**
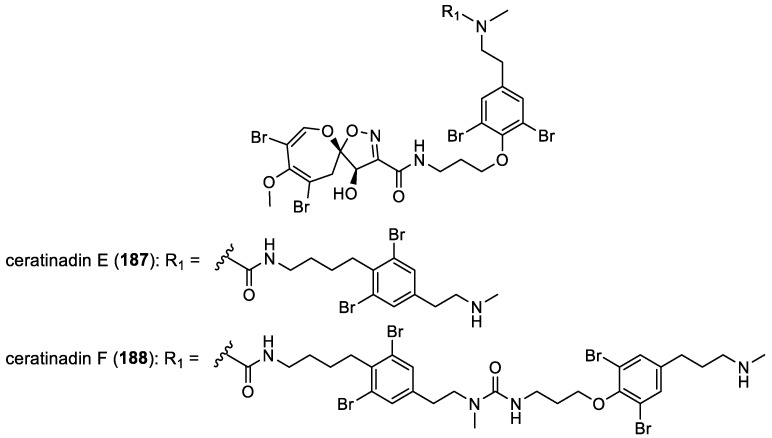
Structures of ceratinadins E (**187**) and F (**188**).

In 2018, ceratinadin E (**187**) and F (**188**) were isolated from the sponge *Pseudoceratina* sp. collected in Okinawa, Japan [30,31]. Ceratinadins E (**187**) and F (**188**) constitute the only two examples of spirooxepinisoxazolines with a bromotyrosine unit (Figure 24). The absolute configurations of their stereogenic centers were determined by a comparison of the NMR and ECD data with those of psammaplysin A (**154**) (Figure 22).

#### 2.2.4. Oximes

This class comprises oximes with a histamine unit, a bromotyramine unit, a disulfide bond or other, rarer unit(s).

- Tyrokeradines A–D

**Figure 25:**
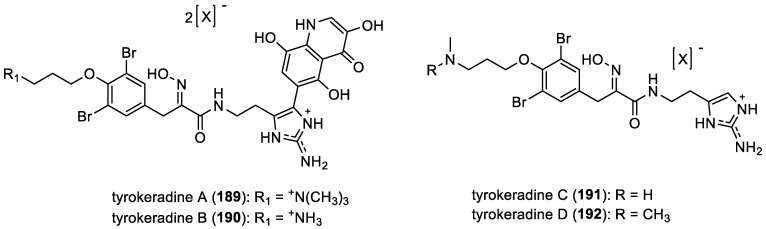
Structures of tyrokeradines A–D (**189**–**192**).

Tyrokeradines A–D (**189**–**192**) were isolated from sponges belonging to the order Verongida collected off Kerama Islands, Okinawa (Figure 25) [32]. These derivatives share a common histamine subunit. Tyrokeradines A (**189**) and B (**190**) feature an *N*-imidazolyl-quinolinone motif, also observed in the structures of ceratinadins A (**126**), B (**127**), and D (**129**) mentioned earlier in Figure 18.

- Araplysillin VI and purealins C–D

**Figure 26:**
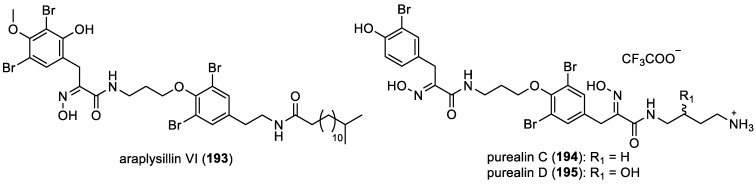
Structures of araplysillin VI (**193**) and purealins C–D (**194**–**195**).

Araplysillin VI (**193**) was isolated by L. Mani et al. from the sponge *Suberea ianthelliformis* collected in the Solomon Islands [67]. It falls into the category of bromotyramine oximes. Additionally, within the same subclass, purealins C (**194**) and D (**195**), along with aplysamine-7 (**56**) (Figure 8), were isolated from *Pseudoceratina* sp. These compounds were obtained as trifluoroacetic acid (TFA) salts through the purification process (Figure 26) [23].

- Psammaplin A and its derivatives

**Figure 27:**
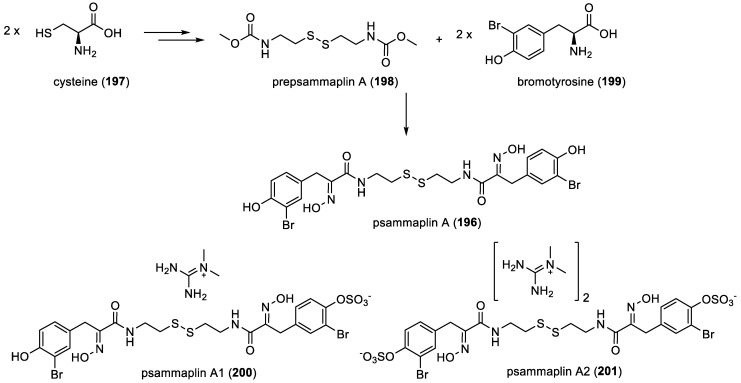
Proposed biosynthesis of psammaplin A (**196**) by H. Niemann et al. [87].

Arabshahi and Schmitz first uncovered psammaplin A (**196**) in an unidentified *Verongida* sponge back in 1987 [40]. More recently, in 2020, E. T. Oluwabusola et al. re-isolated psammaplin A (**196**) from the Fijian marine sponge *Aplysinella rhax* [41]. Psammaplin A (**196**) marked a notable achievement as the first bromotyrosine derivative identified featuring two identical subunits linked by a disulfide bond, each constituted by a bromotyrosine (**198**) and a cysteine-derived fragment named prepsammaplin A (**199**) (Figure 27) [85]. Psammaplin A1 (**200**) and A2 (**201**), water-soluble derivatives, are converted into the more lipophilic psammaplin A (**196**) after an injury of the sponge. This transformation is believed to be mediated by enzymes [86].

**Figure 28:**
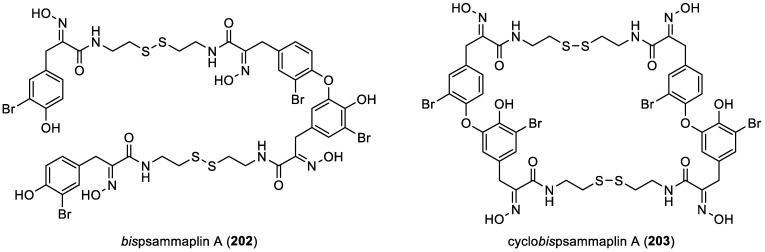
Structures of *bis*psammaplin A (**202**) and cyclo*bis*psammaplin A (**203**).

In 2008, *bis*psammaplin A (**202**), a previously reported compound [57], was found to be a precursor of cyclo*bis*psammaplin A (**203**). Both were isolated from the association of two sponges of the order Tetractinellida: *Jaspis* sp. and *Poecillastra* sp. [58]. All these compounds are represented in Figure 28.

- Pseudoceroximes A–E and pseudoceramines A–D

**Figure 29:**
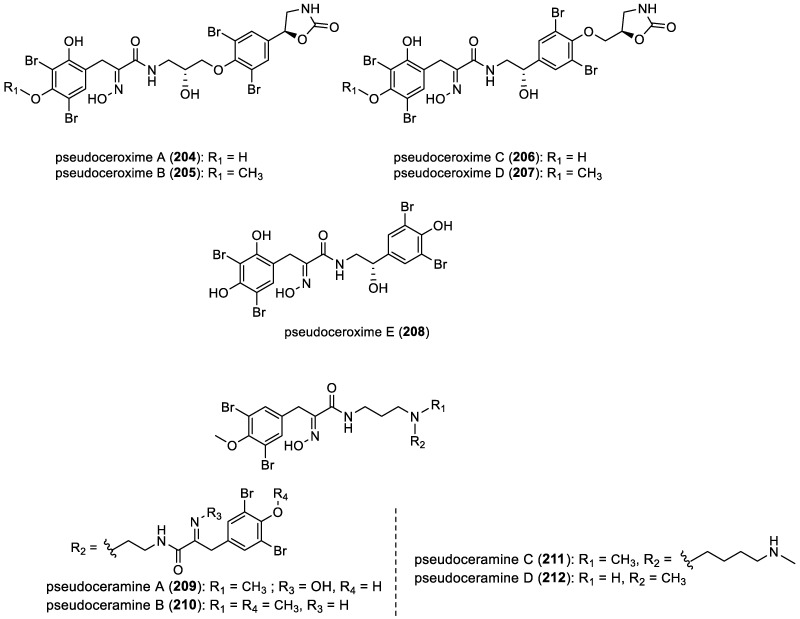
Structures of pseudoceroximes A–E (**204**–**208**) and pseudoceramines A–D (**209**–**212**).

Pseudoceroximes A–E (**204**–**208**) were isolated from *Pseudoceratina* sp. collected in the South China Sea. Pseudoceroximes A–D (**204**–**207**) represent the first examples of the oxime-type bromotyrosine derivatives featuring a 2-oxazolidone ring [52]. Pseudoceramines A–D (**209**–**212**) were isolated from the marine sponge *Pseudoceratina* sp. collected from Great Barrier Reef, Australia (Figure 29) [27].

#### 2.2.5. Bastadin Derivatives

**Figure 30:**
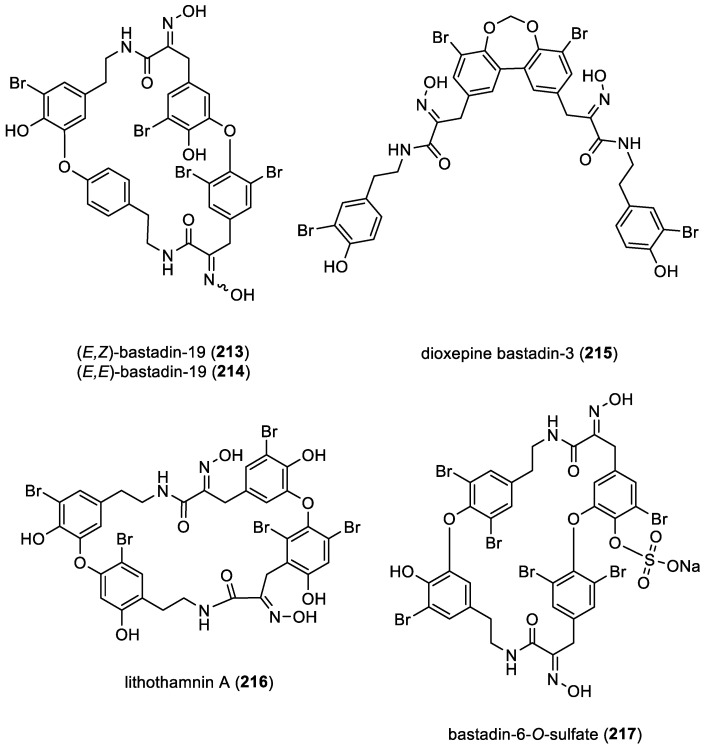
Structures of bastadins-19 (**213**–**214**), dioxepine bastadin-3 (**215**), lithothamnin A (**216**), and bastadins-6-*O*-sulfate (**217**).

Bastadins are composed of two brominated tyrosine and two brominated tyramine units. Since 2004, only two new bastadins, (*E*,*Z*)-bastadin-19 (**213**), a diastereoisomer of (*E*,*E*)-bastadin-19 (**214**), and dioxepine bastadin-3 (**215**), have been reported from the marine sponge *Ianthella reticulata* collected in Milne Bay, Papua New Guinea [88]. Dioxepine bastadin-3 (**215**) is particularly notable for featuring an uncommon dibenzo-1,3-dioxepine functionality, a rare scaffold in natural products chemistry, to date observed only in cercosporins isolated from the terrestrial fungus *Cercospora* spp. [89]. In 2011, lithothamnin A (**216**) was isolated from the red alga *Lithothamnion fragilissimum*. This derivative features a novel aromatic substitution pattern with a *meta*–*meta* linkage between the aromatic rings and a *meta*–*para* linkage also observed in bastadins [90]. In 2018, bastadin-6-*O*-sulfate ester (**217**) was isolated from methanol extracts of the marine sponge *Ianthella basta* (Figure 30) [91].

**Figure 31:**
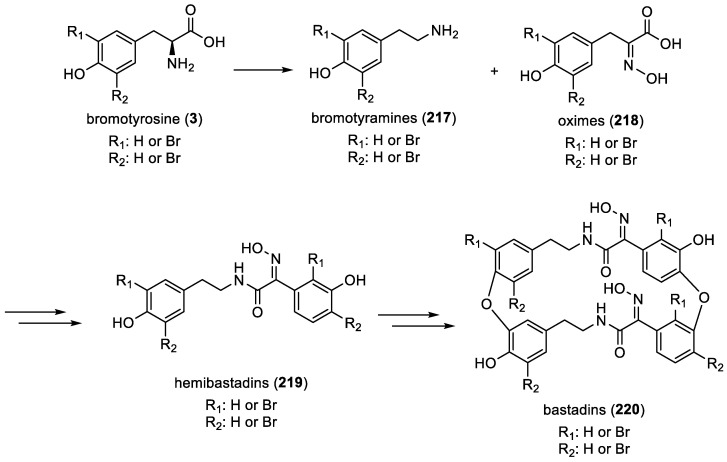
Proposed biosynthesis of bastadins by H. Niemann et al. [87].

Bastadin biosynthesis involves intricate enzymatic processes that result in the formation of these bromotyrosine derivatives. Indeed, the chemical process involves the peptidic condensation of amino acids, followed by the oxidation of the *α*-amino units to yield oxime (**218**) functionalities. This sequence of reactions produces crucial intermediates, hemibastadins (**219**), in bastadin (**220**) biosynthesis. The dimerization of hemibastadins (**219**) can occur through direct carbon-carbon aryl linkages or aryl ether bonds, leading to the formation of either open-chain or macrocyclic bastadins (**220**) [87]. Among these, macrocyclic bastadins are more frequently encountered (Figure 31).

Furthermore, the potential symbiotic origin or involvement of symbionts in the biosynthesis of bastadin-type derivatives (**218**) is supported by the presence of genes encoding flavin-dependent halogenases. These genes sourced from sponge symbionts belonging to *Aplysina cavernicola* and *Ianthella basta* facilitate bromine incorporation into their aromatic structures [72]. In addition, the occurrence of bastadin-like metabolites such as lithothamnin A (**216**) from the red alga *Lithothamnion fragilissimum* contributes to the possibility of symbiotic sources or symbionts playing a role in the biosynthesis of these compounds (Figure 31) [90].

#### 2.2.6. Other Derivatives

**Figure 32:**
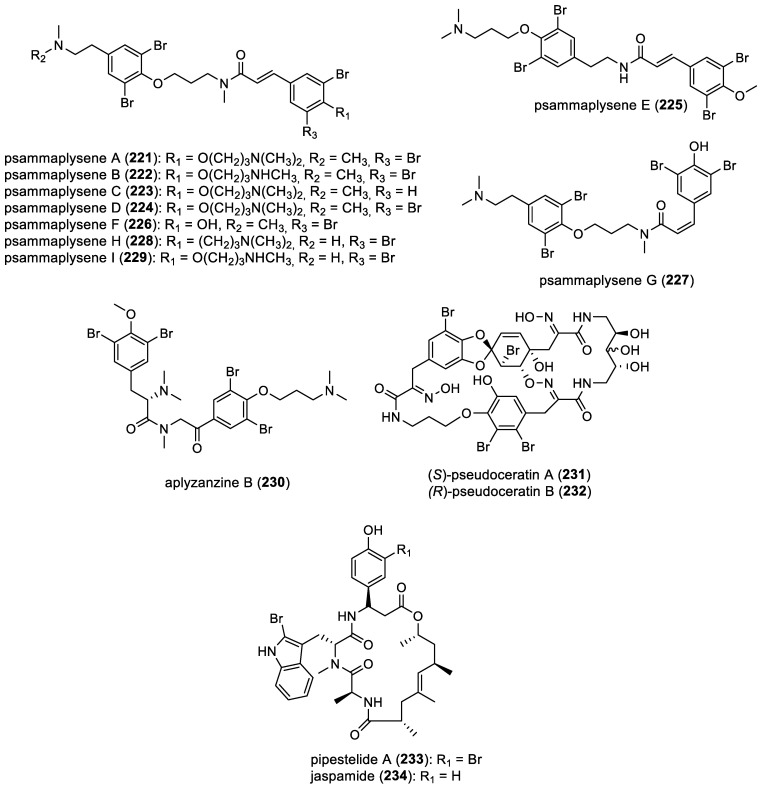
Structures of other bromotyrosine derivatives.

In 2004, psammaplysenes A (**221**) and B (**222**) were isolated from *Psammaplysilla* sp. [92]. In 2007 psammaplysene C (**223**) and D (**224**) were isolated from *Psammoclemma* sp. [93]. In 2017, the examination of the CH_2_Cl_2_/MeOH extract from the Madagascan sponge *Amphimedon* sp. led to the isolation of psammaplysene E (**225**) [92]. More recently, in 2018, psammaplysenes F–I (**226**–**229**) were isolated from the Polynesian sponge *Suberea ianthelliformis* [35]. Aplyzanzine B (**230**) was isolated from a the association *Jaspis* sp. and *Bubaris* sp. collected in Para Island in Indonesia [94]. Pseudoceratins A (**231**) and B (**232**) are cyclic molecules composed of two bromotyrosine subunits, isolated from the sponge *Pseudoceratina purpurea* [95]. Pipestelide A (**233**), a cyclodepsipeptide, seems to be a derivative of jaspamide (**234**) (also known as jasplakinolide) [96]. All compounds are represented in Figure 32.

## 3. Biological Activities

The chemodiversity of bromotyrosines is closely associated with a wide range of biological properties, including antibacterial, antifungal, anticancer, and antiparasitic activities.

### 3.1. Antibacterial Activities

A multitude of bromotyrosine derivatives were evaluated for their antibacterial properties against both Gram-positive (Gram+) bacteria (specifically *Bacillus subtilis* and *Staphylococcus aureus*) and Gram-negative (Gram−) bacteria (notably *Pseudomonas aeruginosa*, *Escherichia coli*, and *Aeromonas hydrophila*). Bromotyrosine antibacterial activities are summarized in Table 1.

In a general context, it appears that bromotyrosines tend to exhibit higher activity against Gram-positive bacteria compared to Gram-negative bacteria. Specifically, most of the compounds assessed for their effects on *Pseudomonas aeruginosa* (ATCC 9027 and ATCC 10145) were found to be inactive, including (−)-aerophobin-2 (**130**), aplysamine-7 (**56**), (+/−)-purealin (**131**), (−)-purealin B (**123**), purpurealidin B (**105**), subereamolline A (**100**), and subereaphenol B (**39**). Notably, ianthelliformisamines A (**7**), B (**8**), and C (**79**) constitute exceptions, as they demonstrated inhibitory effects on the growth of *Pseudomonas aeruginosa*. The presence of the spermine moiety in ianthelliformisamines A (**7**) and C (**79**) appears to be crucial for their activity. Notably, ianthelliformisamine B (**8**), which contains a spermidine moiety instead of spermine, demonstrates a significant reduction in activity, suggesting the importance of the spermine moiety for activity against *P. aeruginosa* [18].

Similarly, among the compounds evaluated, only purpurealidin B (**105**) exhibited activity against *Escherichia coli* (ATCC 25922 and ATCC 11775), whereas pseudoceratin A (**231**) and B (**232**) displayed moderate activity (IZ 7 mm at 10 µg/disk). The remaining compounds, (−)-aerophobin-2 (**130**), aplysamine-7 (**56**), aplysamine-8 (**58**), ceratinadin D (**131**), (+/−)-purealin (**131**), (−)-purealin B (**123**), purpurealidin B (**105**), and tyrokeradine G (**73**) and H (**74**), were found to be inactive against *Escherichia coli*.

In the case of *Aeromonas hydrophila*, 11-*N*-methyl-moloka’iamine (**30**), 11-*N*-cyano-11-*N*-methylmoloka’iamine (**22**), and kuchinoenamine (**29**) exhibited only moderate activity (ZI 8.0 mm at 100 µg), while subereamolline A (**100**) and subereaphenol B (**39**) demonstrated no activity. Furthermore, when tested against *Y. pseudotuberculosis*, pseudoceramines A–D (**209**–**212**) displayed minimal effects, with pseudoceramine B (**210**) showing a moderate effect with an IC_50_ of 40 µM. Lastly, purpurealidin B (**105**) was examined for its effects on various bacteria, including *Klebsiella* sp. (no observed effect), *Vibrio cholerae* (average IC_50_ of 25 µM), and *Shigella flexneri* (weak inhibitory effect with an IC_50_ of 100 µM).

Conversely, several bromotyrosines exhibit activity against *Staphylococcus aureus* (ATCC 25923, ATCC 43300, and ATCC 6538P). These active compounds include ianthelliformisamines A (**7**) and C (**79**), pseudoceratins A (**206**) and B (**207**), (+/−)-purealin (**131**), purealin C (**178**), purpurealidin B (**105**), pseudocerolide C (**49**) and E (**51**), pseudoceroximes A (**204**) and B (**205**), subereamolline A (**100**), subereaphenol B (**39**), and tyrokeradine B (**190**). However, exceptions to this trend were observed with aerophobin-2 (**132**), aplysamine-7 (**56**), aplysamine-8 (**58**), (−)-purealin B (**123**), and tyrokeradines A (**189**), G (**73**), and H (**74**), which did not show activity. Establishing structure-activity relationships for bromotyrosines can be challenging due to the complex and varied outcomes.

Purpurealidin B (**105**) was inactive against *P. aeruginosa* (ATCC 10145) but exhibits an IC_50_ of 10 µM against *S. aureus* (ATCC 25923). Similarly, most of the compounds were active against *Bacillus subtilis* (ATCC 6051 and ATCC 6633), including (−)-aerophobin-2 (**130**), pseudoceratins A (**231**) and B (**232**), (+/−)-purealin (**131**), and (−)-purealin B (**123**). However, aplysamine-7 (**56**) and tyrokeradines G (**73**) and H (**74**) were exceptions as they did not exhibit activity against *Bacillus subtilis*.

Pseudoceramines A–D (**209**–**212**) were evaluated for the inhibition of toxin secretion by the type III secretion (T3S) pathway in *Yersinia pseudotuberculosis*. Among these, pseudoceramine B (**210**) showed a weak inhibition of *Yersinia pseudotuberculosis* outer protein YopE secretion (IC_50_ = 19 µM) and enzymatic activity of YopE (IC_50_ = 19 µM). Additionally, they exhibited a moderate inhibition of YopH enzymatic activity (IC_50_ = 33 µM).

### 3.2. Antifungal Activities

Fourteen bromotyrosine derivatives were evaluated against various fungal species, with a particular focus on *Candida albicans* and *Cryptococcus neoformans*. Additionally, other fungi such as *Saccharomyces cerevisiae*, *Penicillium chrysogenum*, *Mortierella ramanniana*, *Aspergillus fumigatus*, *Aspergillus niger*, *Fusarium* sp., *Rhodotorula* sp., and *Trichophyton mentagrophytes* were included to different extents in the assessment. Bromotyrosine antifungal activities are summarized in Table 2.

Several compounds, including (−)-aerophobin-2 (**130**), aplysamine-7 (**56**), (+/−)-purealin (**131**), (−)-purealin B (**123**), and tyrokeradines B (**190**), G (**73**), and H (**74**) were inactive against *Candida albicans*. Furthermore, purpurealidin B (**105**) demonstrated inactivity against all tested fungal strains, including *Aspergillus fumigatus*, *Fusarium* sp., *Cryptococcus neoformans*, *Aspergillus niger*, *Rhodotorula* sp., and *Candida albicans*. Similarly, ceratinadin C (**128**) showed no activity against either *Cryptococcus neoformans* or *Candida albicans*. On the contrary, ceratinadins A (**126**) and B (**127**) exhibited antifungal activity against *Candida albicans*, with minimum inhibitory concentrations (MICs) of 2 and 4 µM, respectively.

Regarding tyrokeradines, a comparison between tyrokeradines G (**73**), H (**74**), A (**189**), and B (**190**) is possible due to their structural similarities. Tyrokeradine A (**189**) was inactive against *Trichophyton mentagrophytes*, *Cryptococcus neoformans*, *Candida albicans*, and *Aspergillus niger*, while tyrokeradine B (**190**) exhibited activity against all of them, with an MIC of 12.5 µM. The primary difference between these two compounds is the presence of a methylamine group in tyrokeradine A (**189**), whereas tyrokeradine B (**190**) contains a primary amine. Finally, tyrokeradines G (**73**) and H (**74**) displayed comparable antifungal activity and were both inactive against *Trichophyton mentagrophytes* and *Candida albicans*. However, they exhibited weak activity against *Aspergillus niger* (IC_50_ = 32 µM). Notably, tyrokeradine G (**73**), which contains a *β*-alanine group, was active against *Cryptococcus neoformans* (IC_50_ = 16 µM), whereas tyrokeradine H (**74**), featuring a pyridinium ring instead, was inactive against *Cryptococcus neoformans*. Of the fifteen bromotyrosines evaluated against *C. albicans*, only pseudoceroxime A (**204**) (MIC = 11.9 µM) and pseudoceroxime B (**205**) (MIC = 13.0 µM) showed significant activity. In contrast, ceratinines J–M (**52**–**55**), 13-ketohemifistularin-3 (**143**), pseudocerolides B–E (**48**–**51**), and pseudoceroximes C–E (**206**–**208**) demonstrated moderate activity, while pseudocerolide A (**47**) was inactive against *C. albicans*.

### 3.3. Cytotoxic Activities

Except for a few compounds that showed no activity, the overwhelming majority of the forty-four bromotyrosines exhibited activities at the micromolar level against cancer cell lines. It is of great significance to note that a select few compounds demonstrate a significant anticancer effect while exhibiting minimal negative impact on non-cancerous cell lines. Bromotyrosine cytotoxic activities are summarized in Table 3.

While the presence of two tyrosine units is considered crucial for cytotoxic activity, there are instances where this pattern is not followed. As a result, compounds such as purpuroceratic acid A (**102**), psammaplin M (**72**), subereamollines A–B (**100**–**101**), and subereaphenols B–C (**39**–**40**), all of which contain only one tyrosine unit, do not exhibit antitumor activity. Likewise, acanthodendrilline (**32**) exhibited limited efficacy against lung cancer cells H292. However, (*E*,*Z*)-bastadin-19 (**213**) and dioxepine bastadin-3 (**215**), which contain four tyrosine units, surprisingly display no antitumor activity. In contrast, cyclo*bis*psammaplin A (**203**) exhibited activity against several tumor cell lines.

Pseudoceralidinone A (**31**) was selectively active against PC3 prostate cancer cells (IC_50_ = 4.9 µM), while it showed no activity against cervical carcinoma cells HeLa. Moreover, 19-hydroxypsammaplysin Z (**182**) and psammaplysins A (**154**) and Z (**181**) displayed activity against HeLa cell lines. Indeed, aplysamine-7 (**56**) represents the exception to this hypothesis, with its oxime moiety displaying lower activity compared to the spirocyclohexadienylisoxazoline moiety. Furthermore, 20-*N*-methylpurpuramine E (**63**) exhibited low cytotoxicity against HeLa S3 cell lines. Only JBIR-44 (**76**) demonstrated activity against HeLa cell lines (IC_50_ = 3.7 µM).

Purpuramines M (**59**) and N (**60**) showed minimal to no impact on the growth of ovarian cancer cells A2780S, its resistant variant A2780SCP5, and glioma U251MG. Among compounds containing two bromotyrosine units, anomoian B (**81**), aplyzanzine B (**230**), psammaplysin X (**184**), and its 13-hydroxy derivative demonstrated significant activity against a large panel of cancer cell lines: lung cancer cells A549 and NCI-H23, colorectal cancer cells HT-29, colon cancer cells HCT-15, prostate cancer cells PC-3, kidney cancer cells ACHN, stomach cancer cells NUGC-3, and breast cancer cells MDA-MB-231.

Psammaplysin G (**155**) has shown activity against both HEK293 and HEpG2 cell lines. This indicates that psammaplysin G (**155**) exhibited a moderate level of inhibitory activity against both cell lines. On the other hand, psammaplysin F (**156**) demonstrated distinct activity patterns. It is active against HEK293, with an IC_50_ of 10.9 µM, and even more active against HEpG2, with an IC_50_ of 3.7 µM. Psammaplysin F (**156**), therefore, exhibited stronger inhibitory activity against both cell lines compared to psammaplysin G (**155**). Furthermore, the effectiveness of psammaplysins’ actions was primarily influenced by the nature of the substituents attached to the terminal amino group of psammaplysins.

However, this is not the case for purealidins T (**109**) and U (**110**), which exhibited relatively low activity (IC_50_ > 10 µM) against colon cancer cells NCI-H23 (IC_50_ = 6.4 and 3.5 µM), as well as colon cancer cells HCT-8, liver cancer cells Bel-7402, stomach cancer cells BGC-823, lung cancer cells A549, and ovarian cancer cells A2780. Sunabedine (**144**) also displays weak activity against mouse cancer cells B16, and aplysinones A–D (**134**–**137**) were inactive against several cell lines.

Aplysamine-6 (**78**) exhibited effectiveness against isoprenylcysteine carboxyl methyltransferase (Icmt), while araplysillin-*N*_20_-formamide (**112**) has IC_50_ values of 1.1 and 3.8 µM against breast cancer cells MCF-7.

Pipestelide A (**233**) and japamide (**234**), each containing only one bromotyrosine unit, demonstrated significant antitumor activity as cyclodepsipeptides, completely inhibiting the growth of oral carcinoma KB cells at a concentration of 10 µM. Notably, the IC_50_ for pipestelide A (**233**) is exceptionally low (0.10 µM). Anomoian E (**84**) and F (**85**) inhibited oral carcinoma KB cells’ growth at a concentration of 10 µM, achieving 100% and 82% inhibition, respectively. Psammaplysenes D (**224**), F (**226**), and G (**227**) displayed inhibitory effects ranging from 100 to 73% inhibition at 10 µM and from 95 to 17% inhibition at 1 µM.

Additionally, psammaplysenes demonstrated a 90% inhibition of DNA methyltransferase 1 DNMT1 enzyme activity. Pseudoceroximes B (**205**) and D (**207**) displayed moderate antiproliferative effects against U251 cell lines, with IC_50_ values of 14.1 and 20.5 µM, respectively. Additionally, they exhibited similar activity against U87MG cell lines, with IC_50_ values of 17.7 and 25.3 µM, respectively.

### 3.4. Antiparasitic Activities

Twelve out of the twenty-one compounds evaluated for their antiparasitic activities exhibited notable efficacy, as summarized in Table 4. They were evaluated against chloroquine-resistant *Plasmodium falciparum* strains FcB-1 and/or Dd2, as well as susceptible 3D7. Additionally, fistularin-3 (**139**), 19-deoxyfistularin-3 (**140**), purpurealidin B (**105**), 11-hydroxyaerothionin (**138**), (3,5-dibromo-4-hydroxyphenyl)-*N*,*N*,*N*-trimethylethan-1-aminium (**45**), and 2-(3,5-dibromo-4-methoxyphenyl)-*N*,*N*,*N*-trimethylethan-1-aminium (**46**) were tested against *Leishmania panamensis*, *Plasmodium falciparum*, and *Trypanosoma cruzi*.

Among the tested compounds, psammaplysin H (**157**) exhibited the most promising results with an IC_50_ of 0.41 µM against the chloroquine-sensitive *P. falciparum* strain (3D7). Psammaplysin H (**157**) antiparasitic activity was more than 10 times greater than that of psammaplysin G (**155**), also tested in the same study, as well as psammaplysin F (**156**), araplysillin-*N*_20_-formamide (**112**), 19-hydroxypsammaplysin E (**183**), psammaplin A (**196**), and psammaplin D (**70**). Other derivatives, psammaplysins K–M (**160**–**142**), psammaplysin T (**178**), psammaplysin V (**171**), and 19-hydroxypsammaplysin P (**175**), showed no activity. Some compounds exhibited moderate to low levels of activity against Tulahuen C4 strain of *T. cruzi.* These include psammaplin D (**70**) with an IC_50_ of 43 µM, psammaplin A (**196**) with an IC_50_ of 30 µM, fistularin-3 (**139**) with 6% inhibition, and purealidin R (**96**) with 2% inhibition. In the case of the chloroquine-resistant FcB-1 strain, araplysillin-*N*_20_-formamide (**112**) demonstrated activity in the micromolar range (IC_50_ = 3.6 µM). Psammaplysin G (**155**) was less active (with a 98% inhibition of the Dd2 strain). Notably, all isolated compounds from *Verongula rigida* displayed minimal to no activity against the tested parasites.

### 3.5. Other Activities

Several other intriguing biological activities were explored for various bromotyrosine derivatives. For instance, subereaphenols B (**39**) and C (**40**) have exhibited potent antioxidant properties, assessed by using the DPPH assay [37].

In the realm of Alzheimer’s disease research, 3,5-dibromo-4-methoxyphenylpyruvic acid (**9**) has shown promising results. It was found to enhance the secretion of ApoE, a protein associated with Alzheimer’s disease, from astrocytic cancer cells known as CCF-STTG1. These effects were observed at concentrations of 40 µM [10]. Additionally, some bromotyrosine derivatives displayed notable activities related to the inhibition of certain enzymes. Purpuramine M (**59**) exhibited a moderate inhibition (36%) of an enzyme called BACE1, which is implicated in Alzheimer’s disease. Similarly, compounds like araplysillins VII (**118**), IX (**120**), X (**121**), and XI (**122**) demonstrated moderate inhibitory effects (ranging from 35% to 70%) on BACE1 at specific concentrations [45].

Synoxazolidinones A (**33**) and C (**34**), along with pulmonarins A (**35**) and B (**36**), showed notable antifouling activities. Indeed, aplyzanzines C–F (**64**–**67**) were tested against three strains of microalgae (*Porphyridium purpureum*, *Cylindrotheca closterium*, and *Halamphora coffeaeformis*) and three marine bacteria strains (*Vibrio proteolyticus*, *Vibrio aestuarianus*, and *Polaribacter irgensii*) at concentrations up to 10 µM to remain within a high activity range. The most active were aplyzanzine C (**64**) and aplyzanzine E (**66**). Indeed, aplyzanzine C (**64**) is particularly notable as it exhibited a substantial inhibition of both adhesion and growth in marine microalgae, with MIC values at 0.14 µM across all assays [48].

Furthermore, certain bromotyrosine derivatives, psammaplysenes A (**221**) and B (**222**), displayed inhibitory activity in preventing the restoration of FOXO1a, a protein involved in cancer development. These inhibitory effects were observed with IC_50_ values of 5 µM and 20 µM, respectively [30].

Lastly, ianthesin E (**111**) exhibited moderate activity in interfering with the binding between adenosine and its A1 receptor, achieving 61% inhibition at a concentration of 100 µM [56].

## 4. Conclusions

The current review encompasses bromotyrosines that were exclusively isolated from marine organisms within the time frame of 2004 to 2023. A total of 207 new bromotyrosines have been described from 58 different sponge species. It is worth noting that around 47% of these previously unidentified compounds (97 out of 207) have shown diverse bioactivities, with a particular focus on their cytotoxic effects. In fact, 42 of these compounds have demonstrated cytotoxic effects. Notably, there were some compounds among them that exhibited promising or significant bioactivities, which should be given more attention. For instance, there are antibacterial compounds such as suberein-1 and -2, which have shown a minimum inhibitory concentration (MIC) of 0.01 µM against *Vibrio aesturianus*. Additionally, there are antifungal compounds like ceratinadins A and B, which have shown an MIC of 2–8 µM against *Candida albicans* and *Candida neoformans*. Furthermore, there are cytotoxic compounds such as pipestelide A, which has shown an IC_50_ of 0.10 µM against oral carcinoma KB cells. Lastly, there are antiparasitic compounds like psammaplysins F and H, which have shown an IC_50_ of 0.41–1.4 µM, as well as ceratinadin E, which has shown an IC_50_ of 0.77–1.05 µM against *Plasmodium falciparum*. Due to the chemical diversity and biological effects exhibited by these bromotyrosines, further exploration of marine sponges is necessary in order to identify potential lead compounds for the advancement of marine drug development.

## Figures and Tables

**Table 1:** Bromotyrosine antibacterial activities.

Compound	Biological Activity	Ref.
(−)-aerophobin-2 (**130**)	Active against *B. subtilis* (IC_50_ = 2.1 µM) ^a^	[23]
aplysamine-8 (**58**)	Active against *E. coli* (MIC = 125 µM) and against *S. aureus* (MIC = 31 µM)	[53]
ceratinines J–M (**52**–**55**)	Low activity against methicillin-resistant *S. aureus* (MIC > 20 µM) ^b^	[52]
11-*N*-cyano-11-*N*-methylmoloka’iamine (**22**)	Moderate activity against the fish pathogen bacteria *A. hydrophila* (ZI 8.0 mm at 100 µg)	[38]
ianthelliformisamine A (**7**)	Selective activity on *P. aeruginosa*, (MIC = 35 µM) and 77% inhibition of *S. aureus* at 175 µM	[18]
ianthelliformisamine B (**8**)	Minor inhibition of *P. aeruginosa* (80% at 87.5 µM)	[18]
ianthelliformisamine C (**79**)	Activity against *P. aeruginosa* (MIC = 17.5 µM) and *S. aureus* (MIC = 8.75 µM)	[18]
13-ketohemifistularin-3 (**143**)	Low activity against methicillin-resistant *S. aureus* (MIC > 20 µM) ^b^	[52]
kuchinoenamine (**29**)	Moderate activity against pathogen fish bacteria *A. hydrophila* (IZ 8.0 mm at 100 µg)	[38]
11-*N*-methylmoloka’iamine (**30**)	Moderate activity against pathogen fish bacteria *A. hydrophila* (ZI 7.5 mm at 100 µg)	[38]
pseudoceramine B (**210**)	Active against *Y. pseudotuberculosis* (IC_50_ = 40 µM)	[27]
pseudoceratin A (**231**)	Moderate activity against *E. coli* (IZ 7 mm at 10 µg/disk), *B. subtilis* (IZ 7.0 mm at 10 µg/disk), and *S. aureus* (ZI 6.5 mm at 10 µg/disk)	[95]
pseudoceratin B (**232**)	Moderate activity against *E. coli* (IZ 8 mm at 10 µg/disk), *B. subtilis* (IZ 8.0 mm at 10 µg/disk), and *S. aureus* (IZ 7.0 mm at 10 µg/disk)	[95]
pseudocerolide B (**48**)	Low activity against methicillin-resistant *S. aureus* (MIC > 20 µM) ^b^	[52]
pseudocerolide C (**49**)	Active against methicillin-resistant *S. aureus* (MIC = 7.1 µM) ^b^	[52]
pseudocerolide D (**50**)	Moderate activity against methicillin-resistant *S. aureus* (MIC = 12.8 µM) ^b^	[52]
pseudocerolide E (**51**)	Moderate activity against methicillin-resistant *S. aureus* (MIC = 11.2 µM) ^b^	[52]
pseudoceroxime A (**204**)	Active against methicillin-resistant *S. aureus* (MIC = 6.6 µM) ^b^	[52]
pseudoceroxime B (**205**)	Active against methicillin-resistant *S. aureus* (MIC = 5.2 µM) ^b^	[52]
pseudoceroxime C (**206**)	Low activity against methicillin-resistant *S. aureus* (MIC = 17.2 µM) ^b^	[52]
pseudoceroxime D (**207**)	Low activity against methicillin-resistant *S. aureus* (MIC = 1.5 µM) ^b^	[52]
pseudoceroxime E (**208**)	Low activity against methicillin-resistant *S. aureus* (MIC = 10.6 µM) ^b^	[52]
(+/−)-purealin (**131**)	Active against *B. subtilis* (IC_50_ = 2.3 et 3.8 µM) and against *S. aureus* (IC_50_ = 3.8 and 0.83 µM)	[23]
(−)-purealin B (**123**)	Active against *B. subtilis* (IC_50_ = 3.4 and 3.8 µM) ^a^	[23]
purpurealidin B (**105**)	Activity against *E. coli* (IC_50_ > 12 µM), *S. aureus* (IC_50_ = 10 µM), and *V. cholerae* (IC_50_ = 25 µM); low activity against *S. flexineri* (IC_50_ = 100 µM)	[19]
subereamolline A (**100**)	IZ 3 mm against *S. aureus* ^c^	[49]
subereaphenol B (**39**)	IZ 5 mm against *S. aureus* ^c^	[49]
suberein-1 (**146**)	Active against *V. aesturianus* (MIC = 0.01 µM) and *R. littoralis* (MIC = 1 µM) ^d^	[76]
suberein-2 (**147**)	Active against *V. aesturianus* (MIC = 0.01 µM) and *E. coli* (MIC = 0.01 µM) ^d^	[76]
tyrokeradine B (**190**)	Low inhibitory activity against *M. luteus* and *S. aureus* (MIC = 25 µM)	[32]

^a^ *S. aureus* (ATCC 25923), *B. subtilis* (ATCC 6051 and 6633), *E. coli* (ATCC 11775), and *P. aeruginosa* (ATCC 10145); ^b^ methicillin-resistant *S. aureus* (ATCC 43300) and *E. coli* (ATCC 25922); ^c^ *S. aureus* (ATCC 6538P), *P. aeruginosa* (ATCC 9027), and *K. pneumoniae* (ATCC 10032); ^d^ *H. aquamarina* (ATCC 14400), *R. littoralis* (ATCC 495666), *V. aestuarianus* (ATCC 35048), and *E. coli* (ATCC 11775).

**Table 2:** Bromotyrosine antifungal activities.

Compound	Biological Activity	Ref.
ceratinadin A (**126**)	Active against *C. neoformans* (MIC = 4 µM) and *C. albicans* (MIC = 2 µM) ^a^	[30]
ceratinadin B (**127**)	Active against *C. neoformans* (MIC = 8 µM) and *C. albicans* (MIC = 4 µM) ^a^	[30]
ceratinines J–M (**52**–**55**)	Low activity against *C. albicans* (MIC > 20 µM) ^b^	[52]
13-ketohemifistularin-3 (**143**)	Low activity against *C. albicans* (MIC > 20 µM) ^b^	[52]
pseudoceratin A (**231**)	Growth inhibition of a mutant of *S. cerevisiae* (IZ 6.5 mm at 10 µg/disk); good activity against *C. albicans* (IZ 8 mm at 10 µg/disk	[95]
pseudoceratin B (**232**)	Growth inhibition of a mutant of *S. cerevisiae* (IZ 6.5 mm at 10 µg/disk; good activity against *C. albicans* (IZ 6.5 mm at 10 µg/disk)	[95]
pseudocerolide B (**48**)	Low activity against *C. albicans* (MIC > 20 µM) ^b^	[52]
pseudocerolide C (**49**)	Low activity against *C. albicans* (MIC > 20 µM) ^b^	[52]
pseudocerolide D (**50**)	Low activity against *C. albicans* (MIC = 16.0 µM) ^b^	[52]
pseudocerolide E (**51**)	Low activity against *C. albicans* (MIC = 19.2 µM) ^b^	[52]
pseudoceroxime A (**204**)	Active against *C. albicans* (MIC = 11.9 µM) ^b^	[52]
pseudoceroxime B (**205**)	Active against *C. albicans* (MIC = 13.0 µM) ^b^	[52]
pseudoceroxime C (**206**)	Low activity against *C. albicans* (MIC = 19.8 µM) ^b^	[52]
pseudoceroxime D (**207**)	Low activity against *C. albicans* (MIC > 20 µM) ^b^	[52]
pseudoceroxime E (**208**)	Low activity against *C. albicans* (MIC > 20 µM) ^b^	[52]

^a^ *C. albicans* (ATCC 90028); ^b^ *C. albicans* (ATCC 10231).

**Table 3:** Bromotyrosine cytotoxic activities.

Compound	Biological Activity	Ref.
acanthodendrilline (**32**)	Activity against lung cancer cells H292 (IC_50_ = 58.5 µM) without cytotoxicity on healthy cells HaCaT (IC_50_ > 400 µM)	[45]
anomoian B (**81**)	Significant activity against lung A549 (IC_50_ = 5.1 µM), colorectal HT-29 (IC_50_ = 3.2 µM), and breast MDA-MB231 (IC_50_ = 5.3 µM) cancer cells	[61]
anomoian C (**82**)	Active against human squamous cell carcinoma KB cancer cells (28% inhibition at 10 µM and 15% inhibition at 1 µM)	[35]
anomoian D (**83**)	Active against human squamous cell carcinoma KB cancer cells (29% inhibition at 10 µM and 17% inhibition at 1 µM)	[35]
anomoian E (**84**)	Active against human squamous cell carcinoma KB cancer cells (82% inhibition at 10 µM and 6% inhibition at 1 µM)	[35]
anomoian F (**85**)	Active against human squamous cell carcinoma KB cancer cells (100% inhibition at 10 µM and 20% inhibition at 1 µM)	[35]
aplysamine-6 (**78**)	IC_50_ = 14 µM against isoprenylcysteine carboxyl methyltransferase Icmt	[64]
aplysinine B (**38**)	Moderate activity against breast cancer cells (MCF-7, IC_50_ = 25.8 µM), human fibroblasts FS4LTM (IC_50_ = 77.5 µM), and squamous cell carcinoma KB31 (IC_50_ = 32.2 µM)	[48]
aplyzanzine B (**230**)	Significant activity against lung A549 (IC_50_ = 6.1 µM), colorectal HT-29 (IC_50_ = 1.6 µM), and breast MDA-MB231 (IC_50_ = 7.8 µM) cancer cells	[61]
agelanesin A (**41**)	IC_50_ = 9.55 µM against the mouse lymphoma cell L5178Y	[50]
agelanesin C (**42**)	IC_50_ = 16.76 µM against the mouse lymphoma cell L5178Y	[50]
araplysillin VII (**118**)	Cytotoxic against cancerous and healthy cell lines	[54]
araplysillin IX (**120**)	Cytotoxic against cancerous and healthy cell lines	[54]
araplysillin-*N*_20_-formamide (**112**)	IC_50_ = 3.8 µM against breast cancer cells MCF-7	[54]
cyclo*bis*psammaplin A (**203**)	Active against cancer cells of the lung A549 (ED_50_ = 1.95 µM), ovary SK-OV-3 (ED_50_ = 1.21 µM), skin SKMEL-2 (ED_50_ = 1.14 µM), nervous system XF-498 (ED_50_ = 2.88 µM), and colon HCT-15 (ED_50_ = 3.82 µM)	[58]
19-hydroxypsammaplysin X (**186**)	Active against cancer cells of the colon HCT-15 (GI_50_ = 3.5 µM), prostate PC-3 (GI_50_ = 2.1 µM), kidney ACHN (GI_50_ = 2.5 µM), breast MDA-MB-231 (GI_50_ = 0.8 µM), stomach NUGC-3 (GI_50_ = 4.0 µM), and lung NCI-H23 (GI_50_ = 3.5 µM)	[37]
19-hydroxypsammaplysin E (**183**)	Active against colorectal cancer cell line HCT-15 (IC_50_ = 3.8 µM), prostate cancer cell line PC-3 (IC_50_ = 1.4 µM), renal cell carcinoma cell line ACHN (IC_50_ = 2.3 µM), breast cancer cell line MDA-MB-231 (IC_50_ = 0.51 µM), gastric cancer cell line NUGC-3 (IC_50_ = 2.3 µM), and non-small cell lung cancer cell line NCI-H23 (IC_50_ = 3.6 µM)	[84]
19-hydroxypsammaplysin Z (**182**)	Active against the triple-negative breast cancer MDA-MB-231 (IC_50_ = 13.2 µM), cervical carcinoma HeLa (IC_50_ = 17.6 µM), and colorectal carcinoma HCT 116 (IC_50_ = 7.0 µM)	[84]
20-*N*-methylpurpuramine E (**63**)	Low cytotoxicity on cervical cancer cells HeLa S3 (IC_50_ = 4.3 µM)	[29]
JBIR-44 (**76**)	Activity against HeLa cervical cancer cells (IC_50_ = 3.7 µM)	[59]
pipestelide A (**233**)	100% inhibition against oral carcinoma KB cells at 10 and 1 µM (IC_50_ = 0.10 µM)	[96]
psammaplysin X (**184**)	Active against cancer cells of the colon HCT-15 (GI_50_ = 3.3 µM), prostate PC-3, (GI_50_ = 2.3 µM), kidney ACHN (GI_50_ = 3.3 µM), breast MDA-MB-231 (GI_50_ = 1.2 µM), stomach NUGC-3 (GI_50_ = 3.5 µM), and lung NCI-H23 (GI_50_ = 6.4 µM)	[37]
pseudoceralidinone A (**31**)	Active against prostate cancer cells PC3 (IC_50_ = 4.9 µM)	[44]
pseudoceroxime B (**205**)	Active at 40 µM on human glioma cells U87MG (IC_50_ = 17.7 µM) and U251 (IC_50_ = 14.1 µM)	[52]
pseudoceroxime D (**207**)	Active at 40 µM on human glioma cells U87MG (IC_50_ = 25.3 µM) and U251 (IC_50_ = 20.5 µM)	[52]
psammaplysene D (**224**)	Active against human squamous cell carcinoma KB cancer cells (100% inhibition at 10 µM and 95% inhibition at 1 µM), and 90% inhibition of DNA methyltransferase 1 DNMT1 enzyme	[93]
psammaplysene F (**226**)	Active against human squamous cell carcinoma KB cancer cells (73% inhibition at 10 µM and 20% inhibition at 1 µM), and 90% inhibition of DNA methyltransferase 1 DNMT1 enzyme	[35]
psammaplysene G (**227**)	Active against human squamous cell carcinoma KB cancer cells (75% inhibition at 10 µM and 17% inhibition at 1 µM), and 90% inhibition of DNA methyltransferase 1 DNMT1 enzyme	[35]
psammaplysin A (**154**)	Active against the triple-negative breast cancer MDA-MB-231 (IC_50_ = 3.90 µM), cervical carcinoma HeLa (IC_50_ = 8.50 µM), colorectal carcinoma HCT 116 (IC_50_ = 5.1 µM), colorectal cancer cell line HCT-15 (IC_50_ = 3.9 µM), prostate cancer cell line PC-3 (IC_50_ = 6.9 µM), renal cell carcinoma cell line ACHN (IC_50_ = 5.1 µM), breast cancer cell line MDA-MB-231 (IC_50_ = 4.3 µM), gastric cancer cell line NUGC-3 (IC_50_ = 3.8 µM), and non-small cell lung cancer cell line NCI-H23 (IC_50_ = 12.4 µM)	[78,84]
psammaplysin E (**183**)	Active against colorectal cancer cell line HCT-15 (IC_50_ = 3.8 µM), prostate cancer cell line PC-3 (IC_50_ = 3.7 µM), renal cell carcinoma cell line ACHN (IC_50_ = 10.3 µM), breast cancer cell line MDA-MB-231 (IC_50_ = 3.9 µM), gastric cancer cell line NUGC-3 (IC_50_ = 4.0 µM), and non-small cell lung cancer cell line NCI-H23 (IC_50_ = 7.0 µM)	[84]
psammaplysin F (**156**)	Active against embryonic kidney cell line HEK293 (IC_50_ = 10.9 µM) and hepatocellular carcinoma cell line HEpG2 (IC_50_ = 3.7 µM)	[80]
psammaplysin G (**155**)	Active against embryonic kidney cell line HEK293 (IC_50_ = 18.7 µM) and hepatocellular carcinoma cell line HEpG2 (IC_50_ = 17.4 µM)	[26,80]
psammaplin X (**184**)	Active against colorectal cancer cell line HCT-15 (IC_50_ = 3.3 µM), prostate cancer cell line PC-3 (IC_50_ = 2.3 µM), renal cell carcinoma cell line ACHN (IC_50_ = 3.3 µM), breast cancer cell line MDA-MB-231 (IC_50_ = 1.2 µM), gastric cancer cell line NUGC-3 (IC_50_ = 3.5 µM), and non-small cell lung cancer cell line NCI-H23 (IC_50_ = 6.4 µM)	[84]
psammaplysin Z (**181**)	Active against the triple-negative breast cancer MDA-MB-231 (IC_50_ = 19.4 µM), cervical carcinoma HeLa (IC_50_ = 22.2 µM), and colorectal carcinoma HCT 116 (IC_50_ = 8.2 µM)	[84]
purealidin T (**109**)	Low activity (IC_50_ >10 µM) against colon HCT-8, liver Bel-7402, stomach BGC-823, lung A549, and ovarian A2780 cancer cells	[65]
purealidin U (**110**)	Low activity (IC_50_ > 10 µM) against colon HCT-8, liver Bel-7402, stomach BGC-823, lung A549, and ovarian A2780 cancer cells	[65]
purpuramine M (**59**)	Growth inhibition of ovarian cancer cells (A2780S, IC_50_ = 20 µM), its resistant variant (A2780SCP5, IC_50_ = 40 µM), and glioma U251MG (IC_50_ = 50 µM)	[54]
purpuramine N (**60**)	Low inhibition (IC_50_ > 50 µM) against the growth of ovarian cancer cells A2780S, its resistant variant A2780SCP5, and glioma U251MG	[54]
sunabedine (**144**)	IC_50_ = 39 µM against mouse cancer cells B16	[74]

**Table 4:** Bromotyrosine antiparasitic activities.

Compound	Biological Activity	Ref.
Araplysillin-*N*_20_-formamide (**112**)	Activity against chloroquine-resistant and -sensitive *P. falciparum* strains: FcB-1 (IC_50_ = 3.6 µM) and 3D7 (IC_50_ = 7 µM) with an IS of 1.4 between FcB-1 and Vero cells	[67]
ceratinadin E (**187**)	Active against drug-resistant *P. falciparum* strain (IC_50_ = 1.05 µM) and drug-sensitive *P. falciparum* strain (IC_50_ = 0.77 µM)	[30]
fistularin-3 (**139**)	Very weak activity against *L. panamensis* (8% inhibition), *P. falciparum* (11% inhibition), and *T. cruzi* (6% inhibition)	[51]
11-hydroxyaerothionin (**138**)	Very weak activity against *P. falciparum* (8% inhibition) and *L. panamensis* intracellular amastigotes (13% inhibition)	[51]
19-hydroxypsammaplysin E (**183**)	Active against 3D7 strains of *P. falciparum* (IC_50_ = 6.4 µM)	[83]
psammaplin A (**196**)	Moderate activity against Tulahuen C4 strains of *T. cruzi* (IC_50_ = 30 µM) and 3D7 strains of *P. falciparum* (IC_50_ = 60 µM)	[41]
psammaplin D (**70**)	Moderate activity against Tulahuen C4 strains of *T. cruzi* (IC_50_ = 43 µM) and 3D7 strains of *P. falciparum* (IC_50_ = 67 µM)	[41]
psammaplysin F (**156**)	Active against *P. falciparum* chloroquine-resistant strain Dd2 (IC_50_ = 1.4 µM) and 3D7 strains of *P. falciparum* (IC_50_ = 0.87 µM)	[26]
psammaplysin G (**155**)	98% inhibition of *P. falciparum* chloroquine-resistant strain (Dd2) at 40 µM; IC_50_ of 5.23 µM against *P. falciparum* chloroquine-sensitive strain (3D7)	[26,81]
psammaplysin H (**157**)	IC_50_ = 0.41 µM against the chloroquine-sensitive *P. falciparum* strain (3D7).	[26,81]
purpurealidin B (**105**)	Weakly active against *L. panamensis* (2% inhibition) and *P. falciparum* (23% inhibition)	[51]
purealidin R (**96**)	Weakly active against *T. cruzi* (2% inhibition) and *P. falciparum* (7% inhibition)	[51]
